# The lncRNA PARylator promotes PARP1 activation and resistance to DNA‑damaging therapy in esophageal squamous cell carcinoma

**DOI:** 10.1186/s40164-025-00739-z

**Published:** 2025-12-31

**Authors:** Teng Fei Qi, Yadong Liu, Jinkun Yang, Yi Meng Yue, Man Man Han, Hongtao Liu, Jing Li, Min Liu, Yuwei Zhang, Jia Hui Kou, Wen Jin Li, Xiaoying Liu, Ting La, Tao Liu, Song Chen, Xu Dong Zhang, Shundong Cang, Liu Teng, Tianli Fan

**Affiliations:** 1 Translational Research Institute of Henan Provincial People’s Hospital, Henan International Joint Laboratory of Non-coding RNA and Metabolism in Cancer, Henan Provincial Key Laboratory of Long Non-coding RNA and Cancer Metabolism, Zhengzhou City Key Laboratory of Long Non-coding RNA and Cancer Metabolism, Academy of Medical Sciences and Tianjian Laboratory of Advanced Biomedical Sciences of Zhengzhou University, Zhengzhou, 450053 China; 2 School of Life Sciences, Zhengzhou University, Zhengzhou, 450001 China; 3 Department of Pharmacology, School of Basic Medicine, Zhengzhou Universaity, Zhengzhou, 450001 Henan China; 4 Henan Province Hypertension Precision Prevention and Control Engineering Research Center, Henan Provincial People’s Hospital, Zhengzhou, 450053 China; 5 Henan Key Laboratory of Stem Cell Differentiation and Modification, Henan Provincial People’s Hospital, Henan University, Zhengzhou, 450003 China; 6 School of Basic Medical Science, Shanxi Medical University, Taiyuan, 030000 China; 7 National-Local Joint Engineering Research Center of Biodiagnosis & Biotherapy, The Second Affiliated Hospital of Xi’an Jiaotong University, Xi’an, 710004 China; 8 Children′s Cancer Institute Australia for Medical Research, University of New South Wales, Sydney, NSW 2750 Australia; 9 Department of Oncology, Henan Provincial International Coalition Laboratory of Oncology Precision Treatment, Henan Provincial Academician Workstation of Non-coding RNA Translational Research, Henan Provincial Peoples Hospital, Zhengzhou University People′s Hospital, Henan, 450003 China; 10 Department of Nephrology, Henan Key Laboratory of Kidney Disease and Immunology, Henan Provincial People′s Hospital, Zhengzhou, 450003 China

**Keywords:** Esophageal squamous cell carcinoma, PARP1, PARylation, DNA repair, LncRNA, PARylator, LINC00885

## Abstract

**Background:**

Outcomes for patients with esophageal squamous cell carcinoma (ESCC) remain poor, partly due to treatment resistance, particularly to DNA-damaging therapies. Poly(ADP‑ribose) polymerase 1 (PARP1) plays a critical role in repairing single‑strand DNA breaks (SSBs). Unrepaired SSBs can be converted into double‑strand breaks (DSBs) during DNA replication, potentially leading to cell death. Genomic amplification of the distal portion of chromosome 3q (3q26-q29) is a frequent copy‑number alteration in ESCC, which harbors genes encoding several oncoproteins. However, whether long noncoding RNAs (lncRNAs) from this region contribute to ESCC pathogenesis and treatment resistance remains poorly understood.

**Methods:**

In situ hybridization and qPCR were used to assess RNA expression. Protein PARylation was evaluated by immunoprecipitation followed by Western blotting. Cellular phenotypes were quantified using the cell counting kit-8, Annexin V/Propidium iodide staining, and clonogenic assays. DNA damage was monitored by immunofluorescence staining for phosphorylated histone H2AX (γH2AX) and p53-binding protein 1 (53BP1) and by comet assays. RNA-protein interactions were assessed through RNA pulldown coupled with mass spectrometry and RNA immunoprecipitation. Chromatin fractionation and detergent pre-extraction immunofluorescence were conducted to examine PARP1 chromatin association. ESCC growth and responses to treatments were evaluated using xenograft models.

**Results:**

The lncRNA LINC00885, hereafter referred to as PARylator, was the most upregulated lncRNA encoded within the 3q26-q29 amplicon in ESCC. PARylator was predominantly nuclear and interacted with PARP1. Knockdown of PARylator increased γH2AX and 53BP1 foci and comet tail moment, triggered apoptosis, reduced clonogenicity, sensitized ESCC cells to cisplatin and ionizing radiation. In vivo, PARylator knockdown impaired tumor growth and increased cisplatin sensitivity in ESCC xenografts. Mechanistically, PARylator promoted PARP1 recruitment to chromatin and catalytic activation, thereby increasing PARP1 auto-PARylation and enhancing the PARylation of X-ray repair cross-complementing 1 (XRCC1). PARylator was further upregulated in response to DNA damage.

**Conclusions:**

The DNA damage-responsive, 3q26-q29 amplicon-encoded lncRNA PARylator promotes PARP1‑mediated PARylation and SSB repair, thereby limiting DSB accumulation and supporting ESCC cell survival and resistance to DNA-damaging therapies. Targeting PARylator, alone or in combination with DNA-damaging agents, may represent a novel avenue for ESCC treatment.

**Supplementary Information:**

The online version contains supplementary material available at 10.1186/s40164-025-00739-z.

## Introduction

Poly(ADP‑ribose) polymerase 1 (PARP1) is a major sensor of DNA single‑strand breaks (SSBs) [1–4]. SSBs are commonly generated by endogenous metabolic processes and by environmental insults, such as heat stress and ionizing or ultraviolet radiation, which activate the DNA damage response (DDR), a multifaceted network that detects DNA lesions and coordinates repair to maintain genome integrity and cell survival [5–8]. Upon binding SSBs, PARP1 uses NAD + to catalyze the synthesis of poly(ADP‑ribose) (PAR) chains on itself (auto‑PARylation) and on target proteins, thereby recruiting repair factors including X‑ray repair cross‑complementing protein 1 (XRCC1) [1–4]. Most SSBs are efficiently repaired and are typically not lethal [8, 9]. However, if unresolved, SSBs can be converted into double-strand breaks (DSBs) during DNA replication, leading to replication fork collapse and potential cell death [7–10].

Esophageal squamous cell carcinoma (ESCC) accounts for the majority of esophageal cancer cases in many parts of the world, with a particularly high burden in East Asia and parts of Africa [11–13]. However, despite advances in multimodal therapy, including platinum-based chemotherapy, radiotherapy, and immunotherapy, outcomes for ESCC patients remain poor. The five-year survival rate is approximately 15–30% overall, and generally below 10% for those with metastatic disease [11–13]. This dismal prognosis is closely related to intrinsic and acquired treatment resistance, particularly to DNA-damaging therapies [11–14], underscoring the need for a better understanding of the molecular mechanisms that sustain ESCC cell survival under genotoxic stress.

Genomic amplifications in cancer often indicate the presence of oncogenes [15, 16]. The amplification of the distal portion of chromosome 3q (3q26-q29) is among the most frequent copy number alterations in squamous cell carcinomas (SCCs), including ESCC [17–19]. This locus encodes several oncoproteins, such as the p110α subunit of PI3K, SRY-Box Transcription Factor 2 (SOX2), p63, and eukaryotic translation initiation factor 4 gamma 1 (eIF4G1) [17, 20, 21]. However, whether noncoding RNAs from this region play a role in ESCC pathogenesis and treatment resistance remains to be better understood.

Here, we identify the long noncoding RNA (lncRNA) LINC00885, which we refer to as PARylator, as the most upregulated lncRNA encoded within 3q26-q29 in ESCC cells and show that forkhead box protein A1 (FOXA1)-driven transcription contributes to its upregulation. We demonstrate that PARylator binds to PARP1, promotes its chromatin recruitment and activation, enhances SSB repair, limits DSB accumulation, and thus preserves genome integrity. PARylator is inducible by DNA damage, promotes ESCC growth, and confers resistance to cisplatin (CDDP) and ionizing radiation (IR). These findings highlight a PARylator-centered mechanism that augments PARP1-dependent DNA repair and suggesting PARylator as a potential target to sensitize ESCC to DNA‑damaging therapies.

## Methods

### Cell culture and human tissues

The human ESCC cell lines KYSE30, KYSE-150, KYSE450, TE13, and ECA109, and the immortalized, nonmalignant human esophageal epithelial line HET-1 A were cultured in RPMI-1640 (VivaCell; #C3010-0500, Shanghai, China) in a humidified incubator at 37 °C with 5% CO_2_. Cell lines were tested for mycoplasma contamination by PCR every three months. Cell line identity was verified by short tandem repeat profiling using the AmpFlSTR Identifiler PCR Amplification Kit (Thermo Fisher Scientific, 4427368; Applied Biosystems) and analyzed with GeneMarker v1.91 (SoftGenetics, LLC). All cell lines were used within 15 passages after thawing. Details of cell lines are provided in Supplementary Table 1. Formalin-fixed, paraffin-embedded (FFPE) ESCC tissue microarrays with paired adjacent normal esophageal tissues were obtained from the Shanxi Cancer Hospital biobank (Taiyuan, China). No identifiable information about patients such as name and social status was provided. This study was approved by Human Research Ethics Committee of Shanxi Cancer Hospital (KY2023042) and was carried out following the principles embodied in the Declaration of Helsinki.

### Antibodies (Abs) and reagents

Information on the antibodies and reagents used in this study is provided in Supplementary Tables 2 and 3, respectively.

### Cell viability

Cells were seeded at 4 × 10^3^ cells per well in 96-well plates overnight before treatment. Cell Counting Kit-8 (CCK8) solution (Med Chem Express, #HY-K0301) was added and incubated at 37 °C for 2 h. The absorbance at 450 nm was recorded using a Varioskan LUX microplate reader (ThermoFisher Scientific).

### Apoptosis

Apoptotic cells were quantified using the FITC Annexin V Apoptosis Detection Kit (BD Biosciences; Cat#556547) according to the manufacturer’s instructions. In brief, cells were resuspended in binding buffer and incubated with Annexin V/propidium iodide (PI) for 15 min at room temperature in the dark before analysis using a flow cytometer (FACSCanto II, BD Biosciences).

### Cell cycle

Cell-cycle distribution was assessed using the Cell Cycle Analysis Kit (Meilunbio; #MA0334, Dalian, China) according to the manufacturer’s instructions as previously described [22, 23].

### Clonogenicity

Colony formation was conducted as described previously [22, 23]. Colony area (percent well area) and staining intensity (integrated density) were quantified using the ImageJ plugin “ColonyArea” in ImageJ.

### Immunohistochemistry (IHC)

IHC on FFPE tissue sections and quantification of staining were performed as previously described [22, 23]. The percentage of positive tumor cells (0–100%) and staining intensity (0–4: 0 = none, 1 = weak, 2 = moderate, 3 = strong, 4 = very strong) were recorded. An immunoreactive score (IRS) was calculated as (% positive × intensity) / 10. The final IRS for each case was determined as the average score from two independent readers, with any discrepancies resolved through joint review.

### Immunofluorescence (IF)

IF was conducted as described previously [22]. Briefly, cells grown on coverslips were fixed with 4% formaldehyde, and then permeabilized using permeabilization buffer (0.1% Triton X-100 in PBS containing 10% BSA) before incubation with primary Ab. After washing, cells were incubated with Alexa Fluor 488-conjugated secondary Ab or CY3-conjugated secondary Ab in the dark. The coverslip was mounted using the Pro-LongTM Glass Antifade Mountant with NucBlue reagent (Thermo Fisher Scientific, #P36981). Images were acquired on a Leica TCS SP8 confocal laser-scanning microscope using identical acquisition settings across conditions and were processed and quantified using ImageJ.

### In situ hybridization (ISH)

ISH was performed using the RNAscope2.5 HD Detection Reagent-BROWN (Advanced Cell Diagnostics; #322310) according to the manufacturer’s instructions as described previously [22, 23]. The percentage of positive cells (0-100%) and staining intensity (0–4; 0 none, 1 weak, 2 moderate, 3 strong, 4 very strong) were recorded. A reactivity score (RS) was calculated as (% positive × intensity)/10. Slides were evaluated by two blinded observers, and the final score was the average of both. Any discrepancies were resolved by joint review.

### Comet assays

The CometAssay Single Cell Gel Electrophoresis Assay Kit was used according to manufacturer’s instructions (Trevigen, #4250-050-K). Briefly, 500 cells (1 × 10^5^ cells/ml) were mixed with low melting-point agarose on slides at 37 °C. After solidifying for 10 min at 4 °C, the slides were immersed in the lysis solution and then in freshly prepared alkaline unwinding solution to permit DNA unfolding followed by electrophoresis (21 V for 30 min). The slides were washed with double distilled H_2_O twice, immersed in 75% ethanol for 5 min, stained with PI. Analysis of comet images was performed using CASPLab: Comet Assay Software Project Laboratory (http://casp.sourceforge.net; RRID: SCR_007249).

### Western blotting

Western blotting was performed as described before [22, 23]. Band intensities were quantified by densitometry using ImageJ. Details of the antibodies used are provided in Supplementary Table 2.

### Immunoprecipitation (IP)

IP was carried out as described previously [22]. Cells were collected with trypsinization and lysed with lysis buffer (20mM Tris-HCl pH 8.6, 100mM NaCl, 20mM KCl, 1.5mM MgCl_2_, 0.5% NP-40, complete™ EDTA-free Protease Inhibitor Cocktail) on ice for 1 h and centrifuged at 16,000 × g for 30 min. After quantification using a BCA protein assay kit (ThermoFisher, #23225), 3 mg of total protein were rotated with antibodies at 4 °C overnight. Protein-antibody complexes were then captured with the PierceTM Protein A/G Agarose (ThermoFisher Scientific, #20421) at 4 °C for 2 h with rotation and beads were then rinsed with wash buffer (25mM Tris, 150mM NaCl, pH 7.2), boiled and subjected to immunoblotting analysis. Details of the antibodies used are provided in in Supplementary Table 2.

### PARP1 enzymatic activity

PARP1 enzymatic activity was measured using a colorimetric PARP1 activity kit (Genmed Scientifics, Arlington, MA; #GMS50116.1) according to the manufacturer’s instructions. Total protein was extracted, quantified, and a fixed amount (20–100 µg) was added to the reaction mixture containing the kit-supplied PARP buffer, activator, and substrate. After incubation at 37 °C for 1 h, absorbance at 405 nm was measured using a Multiskan SkyHigh Microplate Spectrophotometer (Thermo Scientific, #A51119500C). Sample absorbance values were background-corrected using a no-protein control. PARP1 activity is reported as background-corrected absorbance at 405 nm normalized to total protein (relative activity per mg).

### Mass spectrometry (MS)

Proteins captured by RNA pulldown with biotin‑labeled antisense (AS) or sense probes were resolved by SDS-PAGE (10% polyacrylamide) and visualized by Coomassie Brilliant Blue. A band observed exclusively in the PARYLATOR AS‑probe sample was excised for in‑gel digestion and LC–MS/MS analysis (EASY‑nLC 1000 coupled to an LTQ Orbitrap Velos Pro; Thermo Fisher Scientific). Proteins were reduced, alkylated, and trypsin‑digested. Resulting peptides were purified by C18 chromatography, separated on a C18 analytical column using the EASY‑nLC 1000, and analyzed by data‑dependent acquisition with CID for peptide fragmentation. Proteins identified as potential PARylator interactors are listed in Supplementary Tables 4 and 5.

### Inducible ShRNA knockdown

Cell lines with inducible shRNA targeting PARylator were generated using the FH1‑tUTG doxycycline (Dox)‑inducible knockdown vector as previously described [22, 23]. Knockdown was induced with Dox (1 µg/mL) for 48–72 h unless otherwise specified. shRNA oligonucleotide sequences are listed in Supplementary Table 6.

### Plasmids

The FH1‑tUTG plasmid was a gift from A/Professor M. J. Herold (Walter and Eliza Hall Institute of Medical Research, Australia). The pcDNA3.1(+), pGL4.73[hRluc/SV40], and pSin‑3×Flag plasmids were gifts from Professor Mian Wu (Translational Research Institute, Henan Provincial People’s Hospital, Zhengzhou, China). The pEGFP‑C1 plasmid was a gift from A/Professor Yongyan Wu (Department of Otolaryngology, The First Affiliated Hospital of Shanxi Medical University, Taiyuan, China). Packaging plasmids pMDLg/pRRE (Addgene #12251), pMD2.G (Addgene #12259), and pRSV‑Rev (Addgene ##12253) were obtained from Addgene. Expression and reporter constructs pcDNA3.1‑PARylator, pcDNA3.1‑FOXA1, pGL3‑PARylator‑promoter, and pGL3‑PARylator‑promoter‑ΔFOXA1‑BR were synthesized by Sangon Biotech (Shanghai, China).

### Quantitative PCR (qPCR)

qPCR was carried out as previously described [22, 23]. Relative mRNA levels were calculated using the 2^-ΔΔCt method and normalized to 18 S rRNA. Primer sequences are provided in Supplementary Table 7.

### Absolute quantification of RNA

Absolute quantification was performed by qPCR using a standard‑curve approach detailed previously [23]. Primer sequences are listed in Supplementary Table 7.

### RNA sequencing (RNA-seq)

RNA-seq was performed by GENEWIZ (Suzhou, China). For each sample, 1 µg total RNA was used for library preparation. Poly(A) + mRNA was isolated using a magnetic oligo(dT) capture module and converted to sequencing libraries according to the manufacturer’s instructions. Libraries were sequenced on an Illumina NovaSeq 6000 system (RRID: SCR_016387). Reads were quality‑controlled (FastQC; RRID: SCR_014583), aligned to the human reference genome (e.g., GRCh38) with STAR (RRID: SCR_015899), and gene‑level counts were generated (featureCounts; RRID: SCR_012919). Differential expression analysis was performed using the DESeq2 Bioconductor package (RRID: SCR_015687), which models counts with a negative binomial distribution; Wald test statistics and Benjamini-Hochberg adjusted p values were used to define differentially expressed genes (typically FDR < 0.05).

### Chromatin Immunoprecipitation (ChIP)

ChIP was performed using the ChIP Assay Kit (Beyotime; #P2078) according to the manufacturer’s instructions as described before [22, 23]. Details of the antibodies and primers used are provided in Supplementary Tables 2 and 7, respectively.

### Luciferase reporter assays

Cells were co-transfected with pGL3- PARylator -promoter reporters or pGL3-PARylator-promoter-ΔFOXA1-BR reporters together with pGL4.73[hRluc/SV40] reporters expressing the Renilla luciferase. 48 h later, firefly and Renilla luciferase activities were measured using the Dual‑Luciferase Reporter Assay System (Promega; #E1910) with a Varioskan LUX microplate reader (Thermo Fisher Scientific; #VL0L00D0). Firefly luciferase activity was normalized to Renilla luciferase activity (F/R).

### In vitro transcription

In vitro transcription was performed as described previously [22]. Briefly, Plasmids containing a T7 promoter upstream of the insert were linearized downstream of the transcription unit with BstBI (New England biolab; #R0519S), purified, and quantified. Linear DNA was transcribed with the TranscriptAid T7 High Yield Transcription Kit (Thermo Fisher Scientific, #K0441) according to the manufacturer’s instructions. In vitro-transcribed RNAs were 3′‑end labeled with desthiobiotin using the Pierce RNA 3′ End Desthiobiotinylation Kit (Thermo Fisher Scientific; #20163).

### RNA pull-down (RPD)

RPD was conducted as previously described [22, 23]. Details of the antibodies and primers used are provided in Supplementary Tables 2 and 7, respectively.

### RNA Immunoprecipitation (RIP)

RIP was performed using a Magna RIP™ Kit (Millipore; #17–700; Darmstadt, Germany) according to the manufacturer’s instructions as described previously [22, 23]. Details of the antibodies and primers are provided in Supplementary Tables 2 and 7, respectively.

### Chromatin isolation for PARP1 association

All buffers were kept ice‑cold and supplemented immediately before use with EDTA‑free protease and phosphatase inhibitors under RNase‑free conditions. Cells were treated with hydrogen peroxide (H_2_O_2_, 200 µM) in prewarmed media for 10 min, washed with ice‑cold PBS, scraped, pelleted, and sequentially extracted in CSK buffer (10 mM PIPES pH 6.8, 100 mM NaCl, 300 mM sucrose, 3 mM MgCl_2_, 0.5% Triton X‑100). The remaining nuclear pellet was resuspended in nuclease buffer (10 mM Tris‑HCl pH 7.5, 50 mM NaCl, 3 mM MgCl_2_, 1 mM CaCl_2_, 0.2% Triton X‑100), treated with benzonase (50 U, Merck, #9025-65-4) with gentle mixing, and clarified (16,000 × g, 10 min, 4 °C) to yield the chromatin‑bound fraction (supernatant), which was analyzed by SDS-PAGE and immunoblotting for PARP1 and XRCC1.

### Mouse models

Mice were housed under specific pathogen‑free conditions, individually ventilated cages, standard chow and water added with libitum, 21–23 °C, 40–60% humidity, 12 h light/dark. Cells (1 × 10^7^) in 100 µL solution (PBS: Matrigel 1:1) were subcutaneously injected into the dorsal flanks of 4-week-old female BALB/c nu/nu mice (6 mice per group, Shanghai SLAC Laboratory Animal Co. Ltd., China). Tumor growth was measured every 3 days using a caliper by an investigator blinded to group allocation. Mice were randomized to treatment groups when tumors reached ~ 100 mm^3^. Tumor volume was calculated as V = L × W^2^ / 2. Cisplatin (CDDP) diluted in saline was given through intraperitoneal injection (i.p.) (1 mg/kg) every other day for 10 times. Dox was also diluted in saline and given through i.p. (3 mg/kg) every other day for 7 times. Humane endpoints were predefined. Mice were euthanized when tumors reached 2,000 mm^3^, ulcerated, or if body weight loss exceeded 15%, per protocol. Euthanasia was performed by CO_2_ inhalation followed by cervical dislocation. Studies on animals were conducted in accordance with relevant guidelines and regulations and were approved by the Animal Ethics Review Committee of Zhengzhou University (ZZU-LAC20250912 [12]).

### Statistical analysis

Statistical analysis was carried out using GraphPad Prism 10 to assess differences between experimental groups. Differences were analyzed using a two-tailed Student’s t test or a one-way ANOVA followed by Tukey’s multiple comparisons test. *P* values less than 0.05 were considered statistically significant.

## Results

### Gene copy number gain and transcriptional activation by FOXA1 drive PARylator upregulation in ESCC

**Fig. 1:**
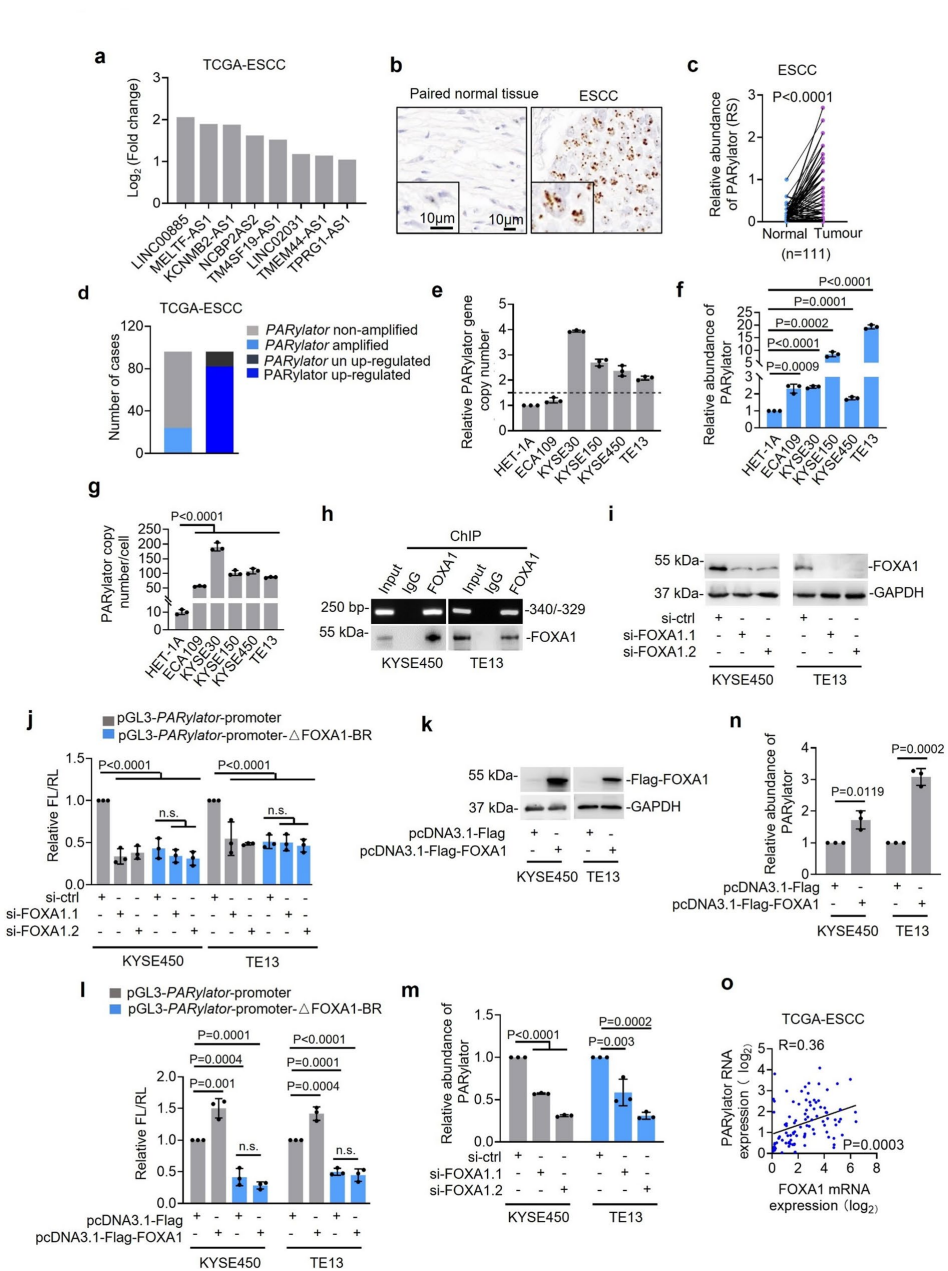
PARylator is upregulated in ESCC cells by genomic amplification and FOXA1-mediated transcription. **a** Analysis of the TCGA-ESCC dataset showing LINC00885 (PARylator) as the most upregulated lncRNA in esophageal squamous cell carcinoma (ESCC) samples (*n* = 82) compared to normal (non-cancerous) esophageal tissues (*n* = 11), among lncRNAs encoded within the distal portion of chromosome 3q (3q26-q29). The top eight upregulated lncRNAs are shown. **b** and **c** Representative microphotographs (b) and quantification (c) of ISH analysis of LINC00885 (PARylator) expression in FFPE ESCC and matched adjacent normal esophageal tissues (*n* = 111 pairs). Data shown are representatives (b) or mean ± s.d. (c); *n* = 3 independent experiments, two-tailed Student’s *t*-test. Scale bar, 10 μm. RS, reactive score. **d** Comparison of the proportion of ESCCs with genomic amplification of the *PARylator* gene (*n* = 24) to those with elevated PARylator expression (*n* = 82) in the TCGA-ESCC cohort (*n* = 96). Two-tailed Student’s t-test. **e** Relative copy number of the *PARylator* gene in the indicated cell lines, measured using qPCR on genomic DNA. Data shown are mean ± s.d.; *n* = 3 independent experiments, one-way ANOVA followed by Tukey’s multiple comparison test. **f** PARylator expression in the indicated ESCC cell lines and the normal esophageal epithelial cell line HET-1 A, as measured by qPCR. Data shown are mean ± s.d.; *n* = 3 independent experiments, one-way ANOVA followed by Tukey’s multiple comparison test. **(g)** Absolute quantification of PARylator by qPCR. Data shown are mean ± s.d.; *n* = 3 independent experiments, one-way ANOVA followed by Tukey’s multiple comparison test. **h** Association of FOXA1 with the FOXA1-BR (-340/-329) at the *PARylator* promoter, measured by ChIP. Data shown are representatives; *n* = 3 independent experiments. **i** and **j** SiRNA knockdown of FOXA1 (i) or deletion of the FOXA1-BR reduced the transcriptional activity of the *PARylator* reporter construct (j), as measured by a dual-luciferase reporter assay. Data shown are representatives (i) or mean ± s.d. (j); *n* = 3 independent experiments, one-way ANOVA followed by Tukey’s multiple comparison test. FL: firefly luciferase; RL: Renilla luciferase. **k** and **l** Overexpression of FOXA1 (k) increased the transcriptional activity of the *PARylator* reporter construct (l), which is reversed by deletion of the FOXA1-BR. Data shown are representatives (k) or mean ± s.d. (l); *n* = 3 independent experiments, one-way ANOVA followed by Tukey’s multiple comparison test. FL: firefly luciferase; RL: Renilla luciferase. **m** SiRNA knockdown of FOXA1 reduced endogenous PARylator expression, as measured by qPCR. Data shown are mean ± s.d.; *n* = 3 independent experiments, one-way ANOVA followed by Tukey’s multiple comparison test. **n** Overexpression of FOXA1 increased endogenous PARylator expression, as measured by qPCR. Data shown are mean ± s.d.; *n* = 3 independent experiments, Two-tailed Student’s t-test. **o** Linear regression analysis of the relationship between PARylator and FOXA1 mRNA expression levels in ESCC samples in the TCGA-ESCC cohort (*n* = 82). Two-tailed Pearson correlation coefficient test

To assess the contribution of noncoding RNAs encoded within distal chromosome 3q (3q26–q29) to ESCC biology, we first asked which lncRNAs from this region are upregulated in ESCC. Analysis of the TCGA squamous esophageal carcinoma subset identified LINC00885 as the most upregulated lncRNA from 3q26–q29 in ESCC versus normal (noncancerous) esophagus (Fig. 1a; Supplementary Fig. 1a). Consistent with the frequent genomic amplification of this locus in SCC, LINC00885 is similarly upregulated in lung squamous cell carcinoma (LUSC) and cervical squamous cell carcinoma (CESC) compared with corresponding normal tissues (Supplementary Fig. 1b, c). Corroborating the upregulation of LINC00885 in ESCC, ISH analysis revealed that LINC00885 expression was often elevated in a panel of 111 FFPE ESCC tissues compared with paired adjacent normal esophageal samples, showing a predominantly nuclear signal (Fig. 1b, c; Supplementary Table 8). Based on these findings, we investigated the functional role of LINC00885 in ESCC cell biology and hereinafter refer to it as “PARylator,” reflecting its ability to promote protein PARylation (see below).

In line with the frequent genomic amplification of the 3q26-q29 region, the *PARylator* locus exhibited copy number gain in 25% of ESCCs in the TCGA-ESCC dataset (Fig. 1d). Nevertheless, this frequency was lower than the proportion of ESCCs with elevated PARylator expression (Fig. 1d), suggesting that additional mechanisms, such as transcriptional regulation, may contribute to the upregulation of PARylator in ESCC. Supporting this notion, qPCR analysis showed that the expression of PARylator was elevated in the ESCC cell line ECA109 lacking copy number gain when compared to the immortalized esophageal epithelial cell line HET-1 A (Fig. 1e, f). Moreover, absolute quantification using qPCR estimated 190 and 56 PARylator molecules per KYSE30 (with copy number gain) and ECA109 (without copy number gain) cell, respectively, compared with approximately 10 PARylator molecules per HET-1 A cell (Fig. 1e, g).

We therefore tested whether transcriptional activation drives PARylator upregulation. Motif analysis of the proximal promoter of the *PARylator* gene predicted a FOXA1-binding site within the proximal promoter (-340 to -329 bp relative to the transcription start site) (Supplementary Fig. 1d). ChIP analysis of TE13 and KYSE450 cells showed FOXA1 enrichment at the predicted FOXA1-binding region (FOXA1-BR) (Fig. 1h). Luciferase reporter assays demonstrated that either FOXA1 knockdown or deletion of the FOXA1 binding region (FOXA1-BR) reduced the activity of the PARylator promoter. (Fig. 1i, j), indicating that this region is required for full promoter activity. Consistently, FOXA1 overexpression increased PARylator reporter activity, which was nevertheless reversed by deletion of the FOXA1-BR (Fig. 1k, l). Indeed, FOXA1 knockdown reduced endogenous PARylator levels (Fig. 1m), while FOXA1 overexpression increased PARylator expression in TE13 and KYSE450 cells (Fig. 1k, n). Moreover, FOXA1 and PARylator expression were correlated in the TCGA-ESCC cohort (Fig. 1o). Collectively, these results indicate that FOXA1-mediated transcriptional regulation, together with genomic amplification, contributes to PARylator upregulation in ESCC cells.

### PARylator promotes ESCC cell survival and proliferation

**Fig. 2:**
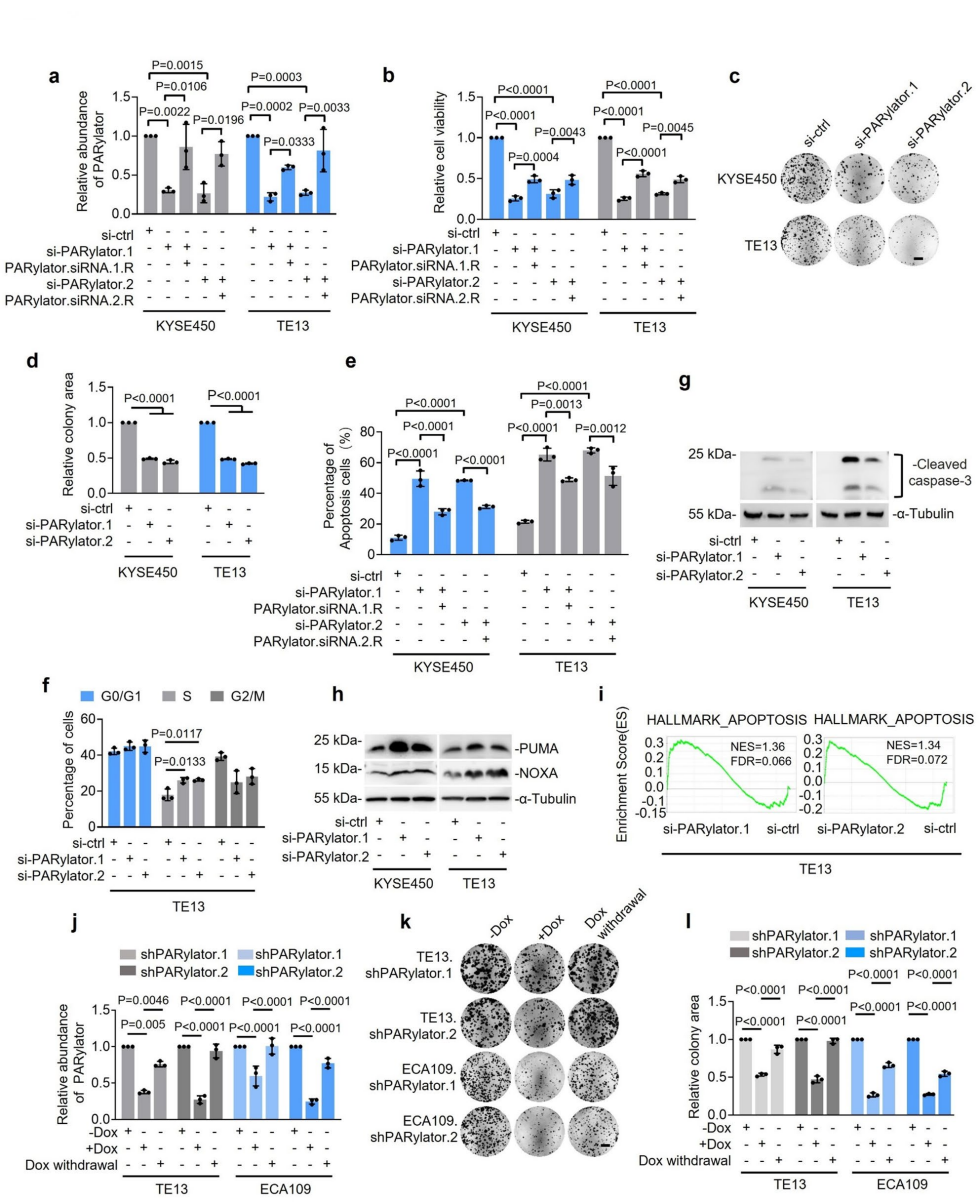
PARylator promotes ESCC cell survival and proliferation. **a** and **b** SiRNA knockdown of PARylator (a) reduced ESCC cell viability (b) as measured using qPCR (a) and CCK-8 assays (b), respectively. Co-transfection of the PARylator siRNA with an siRNA-resistant PARylator expression plasmid rescued both PARylator expression (a) and cell viability (b). Data shown are mean ± s.d.; *n* = 3 independent experiments, one-way ANOVA followed by Tukey’s multiple comparison test. **c** and **d** Representative photographs (c) and quantification (d) of clonogenic assays in ESCC cells with or without siRNA knockdown of PARylator. Data shown are representatives (c) or mean ± s.d. (d); *n* = 3 independent experiments, one-way ANOVA followed by Tukey’s multiple comparison test. Scale bar, 1 cm. **e** SiRNA knockdown of PARylator induced apoptosis in ESCC cells, as measured using flowcytometry analysis of Annexin V/propidium iodide staining, Co-transfection of the PARylator siRNA with an siRNA-resistant PARylator expression plasmid rescued cell apoptosis. Data shown are mean ± s.d.; *n* = 3 independent experiments, one-way ANOVA followed by Tukey’s multiple comparison test. **f** SiRNA knockdown of PARylator caused S phase arrest in TE13 cells, as determined by flowcytometry analysis of propidium iodide staining. Data shown are mean ± s.d.; *n* = 3 independent experiments, one-way ANOVA followed by Tukey’s multiple comparison test. **g** SiRNA knockdown of PARylator induced caspase-3 activation (cleavage), as measured using Western blotting with an anti-cleaved caspase-3 Ab, Data shown are representatives; *n* = 3 independent experiments. **h** SiRNA knockdown of PARylator caused upregulation of PUMA and NOXA, as measured in ESCC cells, as determined by Western blotting. Data shown are representatives; *n* = 3 independent experiments. **i** Gene Set Enrichment Analysis (GSEA) of RNA-seq data from TE13 cells showing that knockdown of PARylator using two independent siRNA caused enrichment of the HALLMARK_APOPTOSIS signature. *n* = 3 experimental repeats. **j** Induced knockdown of PARylator by treatment with doxycycline (Dox, 200 ng/ml), with knockdown effects attenuated upon Dox withdrawal in TE13.shPARylator and ECA109.PARylator cells. Data shown are mean ± s.d.; *n* = 3 independent experiments, two-tailed Student’s *t*-test. **k** and **l** Representative photographs (k) and quantification (l) of clonogenic assays in TE13.shPARylator and ECA109.PARylator cells, with or without induced knockdown of PARylator by treatment with doxycycline (Dox, 200 ng/ml). Cessation of Dox treatment led to restoration of PARylator levels and recovery of clonogenic growth. Data shown are representatives (k) or mean ± s.d. (l); *n* = 3 independent experiments, two-tailed Student’s *t*-test. Scale bar, 1 cm

We next investigated whether upregulation of PARylator confers a growth advantage to ESCC cells. PARylator knockdown reduced viability, as quantified using CCK8 assays, and clonogenicity in TE13 and KYSE450 cells (Fig. 2a-d). These effects were associated with apoptosis, as shown by Annexin V/PI staining (Fig. 2e). In addition, S‑phase accumulation was observed in TE13 cells (Fig. 2f). The inhibition of cell viability and induction of apoptosis were attenuated by ectopic expression of siRNA-resistant PARylator (PARylator.siRNA.1.R and PARylator.siRNA.2.R) (Fig. 2a, b and e), further suggesting that the observed effects are specifically due to the targeted action of PARylator. Notably, the introduction of PARylator siRNA into HET-1 A cells did not significantly affect viability or induce apoptosis (Supplementary Fig. 1e, f and g), which is consistent with the very low expression of PARylator in this cell line (Fig. 1f, g). These results suggest that PARylator is dispensable for survival and proliferation in non-malignant esophageal epithelial cells but functionally important in ESCC cells.

The induction of apoptosis upon PARylator knockdown in TE13 and KYSE450 cells was corroborated by cleaved caspase‑3 detected by Western blotting (Fig. 2g) [24]. Moreover, Western blotting analysis revealed that the knockdown resulted in upregulation of the BH3-only proapoptotic proteins, Phorbol-12-myristate-13-acetate-induced protein 1 (PMAIP1, also known as NOXA) and p53 upregulated modulator of apoptosis (PUMA, also known as BBC3) (Fig. 2h), both of which are commonly induced by DNA damage and contribute to apoptosis [25, 26]. Considering that both TE13 and KYSE450 cells harbor *TP53* mutations, the upregulation of NOXA and PUMA and induction of apoptosis upon PARylator knockdown in these cells likely reflects p53-independent mechanisms. Regardless, gene set enrichment analysis (GSEA) of the RNA-seq data from TE13 cells showed significant enrichment of the HALLMARK_APOPTOSIS signature upon PARylator knockdown with two independent siRNAs (Fig. 2i). Together, these results indicate that PARylator supports ESCC cell survival and proliferation, consistent with engagement of DDR signaling.

To assess reversibility and strengthen specificity, we established sublines of TE13 and ECA109 cells (designated as TE13.shPARylator.1 and TE13.shPARylator.2 as well as ECA109.shPARylator.1 and ECA109.shPARylator.2) with conditional knockdown of PARylator upon treatment with Dox. Induction of PARylator knockdown by Dox treatment (200 ng/ml) led to a reduction in clonogenicity (Fig. 2j-l). Upon doxycycline withdrawal, PARylator levels were restored, and clonogenic growth recovered (Fig. 2j-l). These observations further support the specificity of PARylator shRNAs and reinforce the role of PARylator in promoting ESCC cell survival and proliferation.

### PARylator interacts with PARP1

**Fig. 3:**
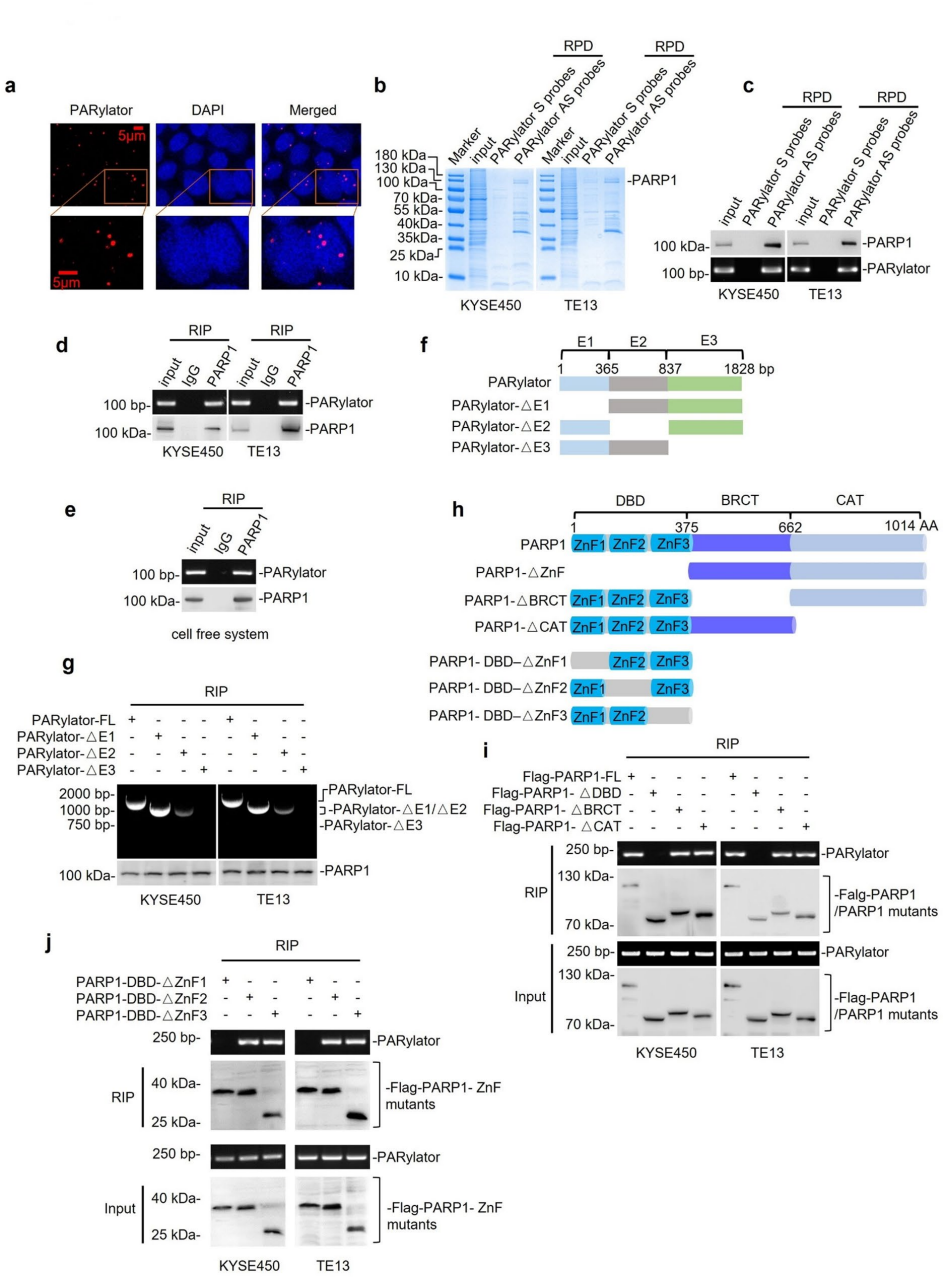
PARylator interacts with PARP1. **a** FISH analysis showing the predominant nuclear localization of PARylator in TE13 cells. Data shown are representatives; *n* = 3 independent experiments. **b** PARP1 was the most abundant protein recovered in PARylator RNA pull-downs, as determined by LC-MS/MS. *n* = 1 experiment. **c** and **d** The interaction between PARylator and PARP1 demonstrated by RPD (c) and RIP (d). Data shown are representatives; *n* = 3 independent experiments. **e** PARylator bound to recombinant PARP1 in a cell-free system, as shown by RIP. Data shown are representatives; *n* = 3 independent experiments. **f** Schematic illustration of full-length PARylator (PARylator‑FL) and the indicated PARylator fragments (E1: 1-365; E2: 366–837; and E3: 838–1828) used for mapping experiments. **g** Deletion of E3 (△838–1828) of PARylator, but not deletion of E1 (△1–365) or E2 (△366–837), abolished the association of PARylator with PARP1. Data shown are representatives; *n* = 3 independent experiments. FL: full-length. **h** Schematic illustration of full-length PARP1 (PARP1‑FL) and the PARP1 mutant with indicated fragment deletions used for mapping experiments. **i** Deletion of the DBD, but not deletion of the BRCT or CAT, abolished PARP1 association with PARylator, as shown in RIP. Data shown are representatives; *n* = 3 independent experiments. **j** Deletion of ZnF1, but not ZnF2 or ZnF3, diminished PARP1 association with PARylator, as shown in RIP. Data shown are representatives; *n* = 3 independent experiments

To confirm the predominant nuclear signal of PARylator observed in the ISH study (Fig. 1b), we carried out FISH analysis of TE13 cells. This analysis similarly demonstrated that PARylator primarily localized to the nucleus (Fig. 3a). To identify PARylator‑associated proteins, we performed RPD followed by mass spectrometry (MS). PARP1 appeared to be the most abundant protein that coprecipitated with PARylator in both TE13 and KYSE450 cells (Fig. 3b; Supplementary Tables 4 and 5), consistent with its role as a nuclear enzyme essential for SSB repair [1–4]. The association between PARylator and PARP1 was further confirmed by RPD and RIP assays in TE13 and KYSE450 cells (Fig. 3c, d). Furthermore, in vitro synthesized PARylator interacted with recombinant PARP1 in a cell-free system as measured by RIP (Fig. 3e), supporting the existence of a direct interaction.

To identify the region of PARylator responsible for binding PARP1, we performed deletion mapping experiments using in vitro-transcribed PARylator mutants (Fig. 3f). RIP analysis revealed that deletion of the segment corresponding to exon 3 (E3, 838–1828) of PARylator, but not deletion of the segment corresponding to E1 (1–365) or E2 (366–837), abolished the association of PARylator with PARP1 (Fig. 3g), indicating that the nucleotides 838–1828 are critical for PARP1 binding. In addition to identifying the region of PARylator responsible for binding PARP1, we also mapped the determinant on PARP1 required for binding to PARylator (Fig. 3h). Deletion of the DNA-binding domain (DBD), but not deletion of the BRCA1 C-terminus (BRCT) or catalytic domain (CAT), abolished the association of PARP1 with PARylator, as evidenced by RIP assays (Fig. 3i). Within the DBD, deletion of zinc finger motif 1 (ZnF1), but not deletion of ZnF2 or ZnF3, markedly reduced the interaction of PARP1 with PARylator (Fig. 3j), indicating that ZnF1 in PARP1 is critical for its association with PARylator.

### PARylator facilitates parylation and limits DSB accumulation

**Fig. 4:**
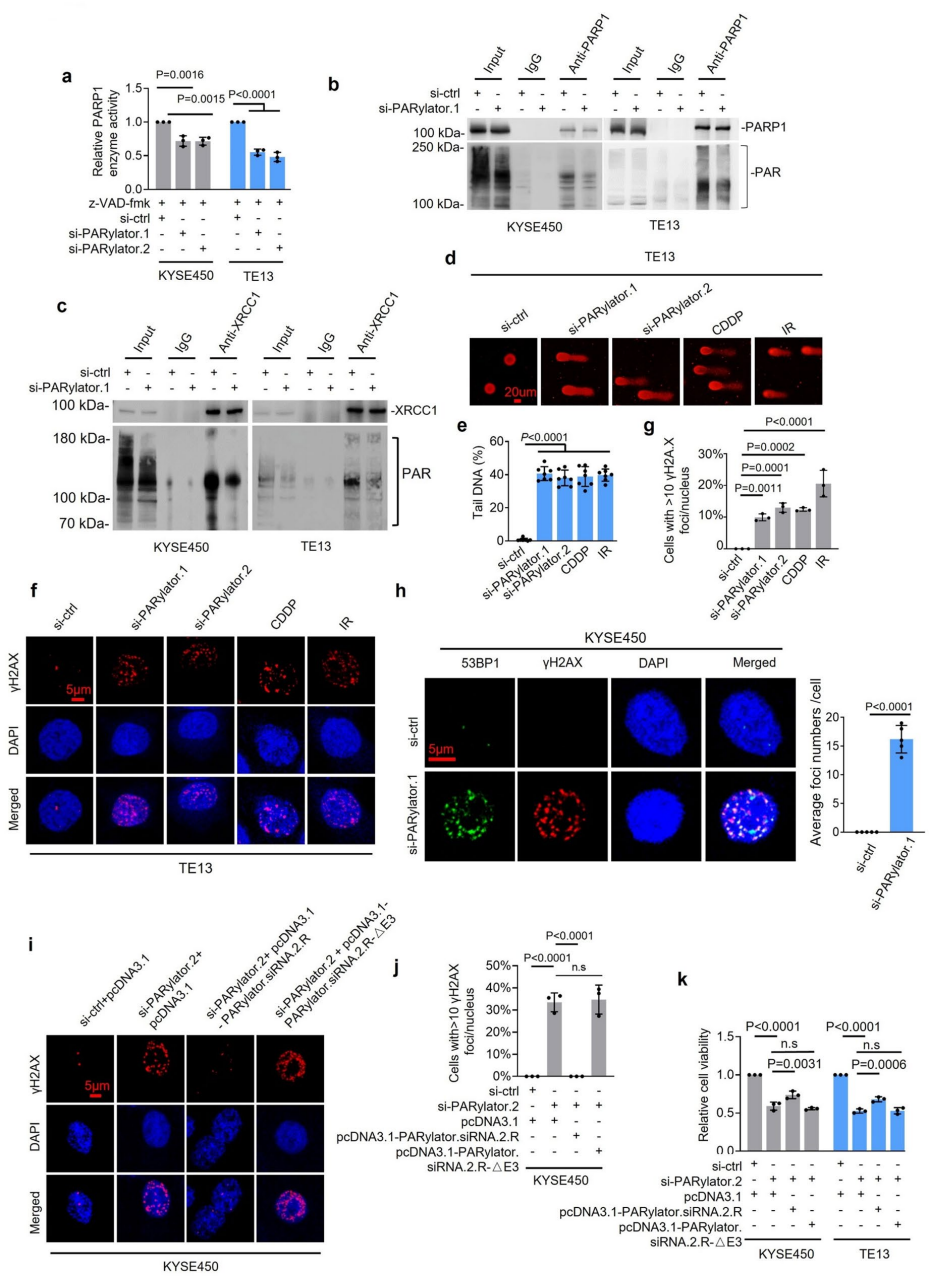
PARylator promotes PARP1 activation. **a** and **b** In TE13 and KYSE450 cells pretreated with the pan-caspase inhibitor z-VAD-fmk (50 µM), siRNA knockdown of PARylator reduced PARP1 catalytic activity (a) and auto-PARylation (b), as measured using colorimetric PARP1 activity assays (a) and immunoprecipitation followed by Western blotting (b), respectively. Data shown are mean ± s.d (a) or representatives (b); *n* = 3 independent experiments, one-way ANOVA followed by Tukey’s multiple comparison test. **c** In TE13 and KYSE450 cells pretreated with the pan-caspase inhibitor z-VAD-fmk (50 µM), siRNA knockdown of PARylator decreased XRCC1 PARylation, as determined by immunoprecipitation followed by Western blotting. Data shown are representatives; *n* = 3 independent experiments. **d** and **e** Representative microphotographs (d) and quantification (e) of comet tails in TE13 cells with or without siRNA knockdown of PARylator. Data shown are representatives (d) or mean ± s.d. (e); *n* = 3 independent experiments, one-way ANOVA followed by Tukey’s multiple comparison test. Scale bar, 20 μm. **f** and **g** Representative microphotographs (f) and quantification (g) of immunofluorescence staining of γH2AX in TE13 cells with or without siRNA knockdown of PARylator. Data shown are representatives (f) or mean ± s.d. (g); *n* = 3 independent experiments, one-way ANOVA followed by Tukey’s multiple comparison test. Scale bar, 5 μm. **h** SiRNA knockdown of PARylator caused the accumulation of 53BP1 foci that colocalized with γH2AX in TE13 cells, as measured by immunofluorescence staining. Data shown are representatives; *n* = 3 independent experiments. Scale bar, 5 μm. **i** and **j** Representative microphotographs (i) and quantification (j) of immunofluorescence staining of γH2AX in KYSE450 cells, showing that expression of an siRNA-resistant PARylator (PARylator.siRNA.2.R), but not a siRNA-resistant PARylator with the E3 fragment (838–1828) deleted (PARylator.siRNA.2.R‑ΔE3), diminished the increase in γH2AX foci caused by siRNA knockdown of endogenous PARylator. Data shown are representatives (i) or mean ± s.d. (j); *n* = 3 independent experiments, two-tailed Student’s *t*-test. Scale bar, 5 μm. **k** Expression of a siRNA-resistant PARylator (PARylator.siRNA.2.R), but not a siRNA-resistant PARylator with the E3 fragment (838–1828) deleted (PARylator.siRNA.2.R‑ΔE3), partially rescued the reduction in viability caused by siRNA knockdown of endogenous PARylator in KYSE450 cells. Data shown are mean ± s.d.; *n* = 3 independent experiments, one-way ANOVA followed by Tukey’s multiple comparison test

We asked whether PARylator modulates PARP1 expression and activity. Since PARylator knockdown induced activation of caspase-3 (Fig. 2g), which is known to proteolytically cleave PARP1 during apoptosis [24], we pretreated TE13 and KYSE450 cells with the pan-caspase inhibitor z-VAD-fmk (50 µM) before knocking down PARylator using siRNAs. Under these conditions, PARylator knockdown did not significantly alter the PARP1 protein levels, determined by Western blotting (Supplementary Fig. 2a). However, PARylator knockdown reduced PARP1 enzymatic activity, quantified by a colorimetric assay for PARP1 activity, and decreased its auto-PARylation, as measured by PARP1 immunoprecipitation followed by anti‑PAR immunoblotting (Fig. 4a, b). In contrast, overexpression of PARylator modestly increased PARP1 activity (Supplementary Fig. 2b, c). Additionally, the PARylation of X-ray repair cross-complementing 1 (XRCC1), a scaffold protein critical for recruiting factors involved in SSB repair [10, 27], was reduced following PARylator knockdown, as determined by anti‑PAR immunoblotting of XRCC1 immunoprecipitates (Fig. 4c). Thus, PARylator promotes PARP1 activation and, consequently, the PARylation of both PARP1 itself and XRCC1.

Unrepaired SSBs can stall or collapse DNA replication forks during the S phase, leading to DSBs [9, 10]. Since PARylator promotes PARP1 activation (Fig. 4a, b and Supplementary Fig. 2b, c), we reasoned that reduced PARP1 activation upon PARylator knockdown would increase DSBs. Supporting this, Western blotting showed that PARylator knockdown triggered phosphorylation of ataxia telangiectasia mutated (ATM; pS1981) and phosphorylation of checkpoint kinase 2 (CHK2; pT68) (Supplementary Fig. 2d). Moreover, PARylator knockdown increased comet tail moments and phosphorylated histone H2AX (γH2AX) foci in TE13 and KYSE450 cells, as measured using IF analysis (Fig. 4d-g and Supplementary Fig. 2e, f). These changes partially mimic the DSB phenotype induced by CDDP (0.25 µg/ml) or IR (4 Gy, single fraction) (Fig. 4d-f and Supplementary Fig. 2e, f), both standard treatments in ESCC management [12, 28]. In addition, IF analysis demonstrated that PARylator knockdown led to the accumulation of p53-binding protein 1 (53BP1) foci that colocalized with γH2AX (Fig. 4h and Supplementary Fig. 2g). These results are consistent with replication‑dependent DSBs and support that PARylator contributes to the maintenance of genomic integrity.

To test whether PARP1 binding is required for these effects, we performed rescue experiments. Expression of a siRNA-resistant PARylator (PARylator.siR) rescued KYSE450 cells from DNA damage and the reduction in cell viability caused by siRNA knockdown of endogenous PARylator, whereas a siRNA‑resistant PARylator lacking the 838–1828 fragment (PARylator.siR‑ΔE3), did not (Fig. 4i-k), indicating that the interaction of PARylator with PARP1 is required for its effects.

### PARylator promotes PARP1 chromatin association

**Fig. 5:**
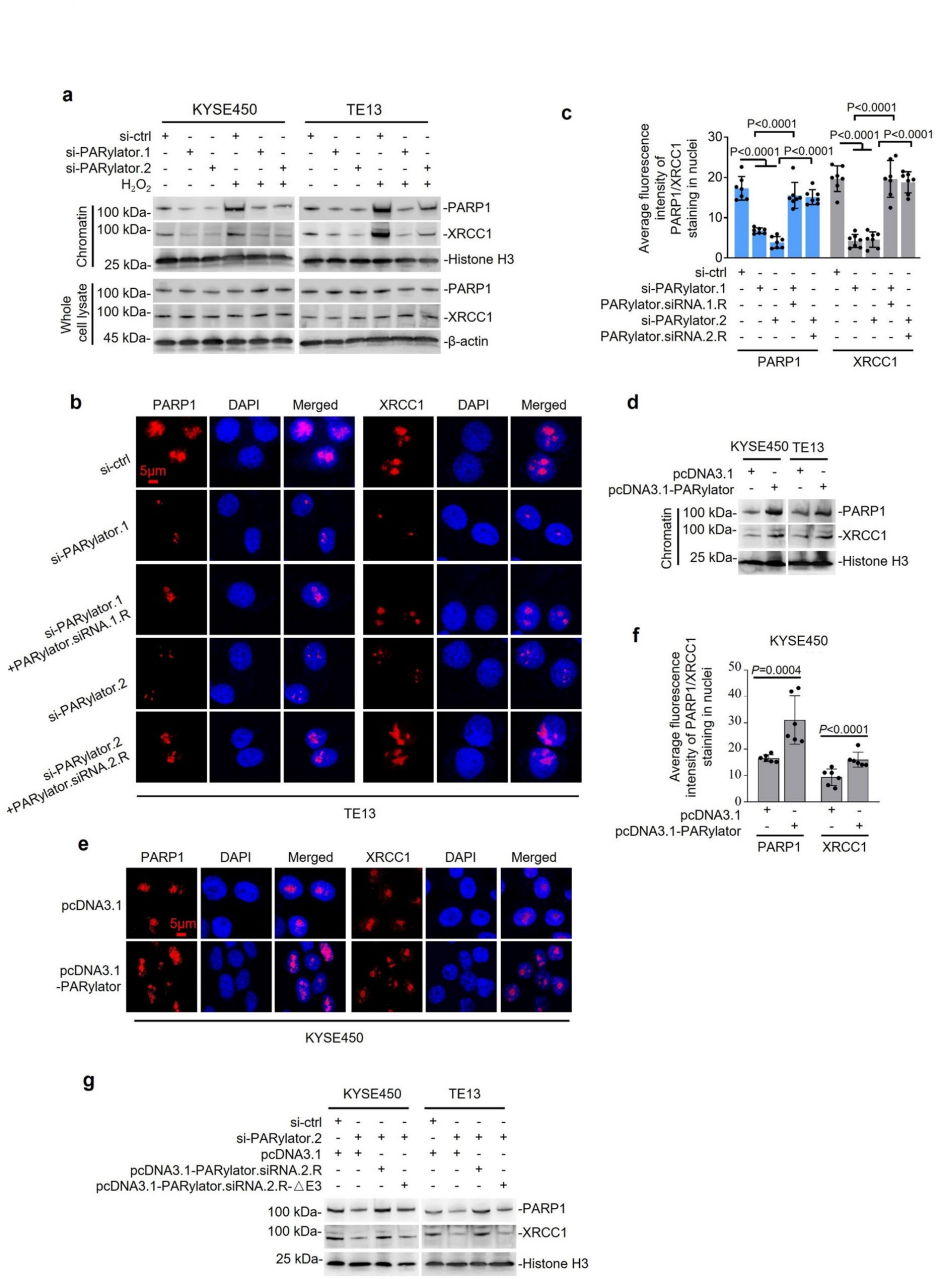
PARylator promotes PARP1 chromatin association. **a** siRNA knockdown of PARylator reduced the association of PARP1 and XRCC1 with chromatin in TE13 and KYSE450 cells treated with or without H_2_O_2_ (200 µM, 1 h), as measured by chromatin fractionation followed by Western blotting. Data shown are representatives; *n* = 3 independent experiments. **b** and **c** Representative microphotographs (b) and quantification (c) of immunofluorescence staining of PARP1 and XRCC1 under detergent pre-extraction conditions in TE13 cells. Cells were subjected to: (i) Control siRNA, (ii) PARylator siRNA, or (iii) PARylator siRNA followed by rescue with an siRNA-resistant PARylator expression plasmid. All groups were treated with H₂O₂ (200 µM, 1 h). Data shown are representatives (b) or mean ± s.d. (c); *n* = 3 independent experiments, one-way ANOVA followed by Tukey’s multiple comparison test. Scale bar, 5 μm. **d** PARylator overexpression increased the association of PARP1 and XRCC1 with chromatin in TE13 and KYSE450 cells treated with H_2_O_2_ (200 µM, 1 h), as determined by chromatin fractionation followed by Western blotting. Data shown are representatives; *n* = 3 independent experiments. **e** and **f** Representative microphotographs (e) and quantification (f) of immunofluorescence staining of PARP1 and XRCC1 under detergent pre‑extraction conditions in KYSE450 cells overexpressing PARylator and treated with H_2_O_2_ (200 µM, 1 h). Data shown are representatives (e) or mean ± s.d. (f); *n* = 3 independent experiments, one-way ANOVA followed by Tukey’s multiple comparison test. Scale bar, 5 μm. **g** Expression of PARylator.siRNA.2.R, but not PARylator.siRNA.2.R ‑ΔE3 (838–1828), increased PARP1 and XRCC1 association with chromatin in ESCC cells with endogenous PARylator knocked down by siRNA, as determined by chromatin fractionation followed by Western blotting. Data shown are representatives; *n* = 3 independent experiments

Because PARP1 is activated upon binding to SSBs on chromatin [10, 29], we sought to determine PARylator influences PARP1 activation by modulating the association of PARP1 with chromatin in ESCC cells. TE13 and KYSE450 cells were transfected with PARylator or control siRNAs for 24 h and then exposed to H_2_O_2_ (200 µM) to induce oxidative SSBs [30]. Measurements were conducted 1 h after the addition of H_2_O_2_, before the onset of significant apoptosis. Chromatin fractionation followed by Western blotting revealed that PARylator knockdown reduced the enrichment of PARP1 in the chromatin fraction in response to H_2_O_2_ treatment (Fig. 5a). Consistently, detergent pre-extraction with 0.2% Triton X-100 for 5 min before immunofluorescence showed that PARylator knockdown decreased PARP1 retention on chromatin (Fig. 5b, c and Supplementary Fig. 3a, b). Nevertheless, this decrease in PARP1 retention was attenuated by ectopic expression of siRNA-resistant PARylator (PARylator.siRNA.R) (Fig. 5b, c). PARylator knockdown also diminished chromatin-associated XRCC1 (Fig. 5a-c and Supplementary Fig. 3a, b) in TE13 and KYSE450 cells. Conversely, PARylator overexpression modestly increased the levels of chromatin-associated PARP1 and XRCC1 (Fig. 5d-f and Supplementary Fig. 3c, d). Expression of a siRNA-resistant PARylator (PARylator.siR), but not a PARP1-binding-deficient mutant (PARylator.siR‑ΔE3), partially restored PARP1 chromatin enrichment in PARylator-depleted ESCC cells (Fig. 5g), indicating that PARylator-PARP1 interaction is critical for efficient PARP1 chromatin association.

### PARylator is responsive to DNA damage

**Fig. 6:**
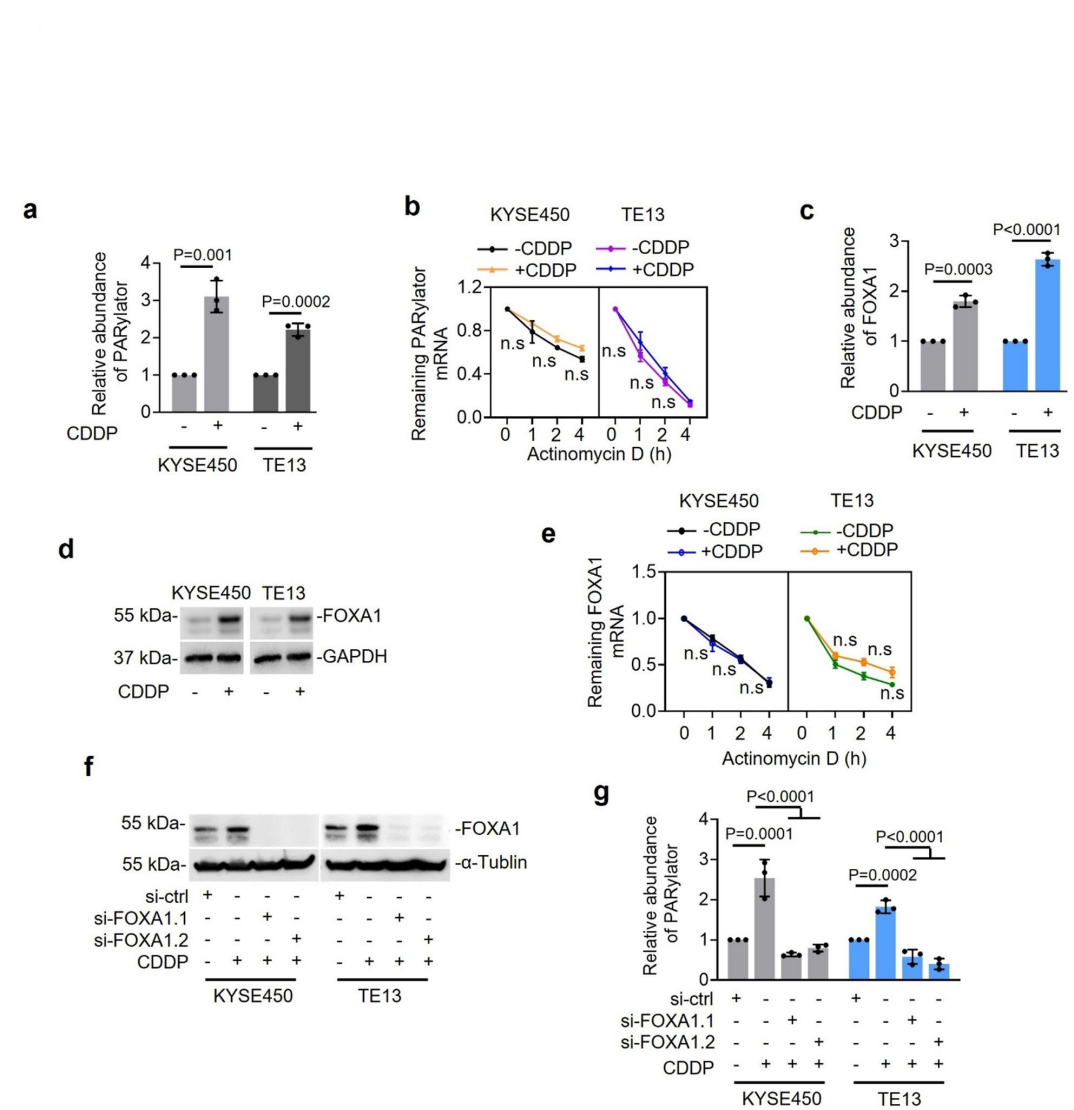
Treatment with CDDP upregulates PARylator. **a** Treatment with CDDP (0.5 µg/ml, 24 h) elevated PARylator levels in TE13 and KYSE450 cells, as measured by qPCR. Data shown are mean ± s.d.; *n* = 3 independent experiments, two-tailed Student’s *t*-test. **b** Treatment with CDDP (0.5 µg/ml, 24 h) did not alter the half-life of PARylator in TE13 and KYSE450 cells, as measured by ActD-chase assays using qPCR. Data shown are mean ± s.d.; *n* = 3 independent experiments, two-tailed Student’s *t*-test. **c** and **d** Treatment with CDDP (0.5 µg/ml, 24 h) increased FOXA1 mRNA (c) and protein (d) expression in TE13 and KYSE450 cells, as measured by qPCR (c) and Western blotting (d), respectively. Data shown are mean ± s.d. (c) or representatives (d); *n* = 3 independent experiments, two-tailed Student’s *t*-test. **e** Treatment with CDDP did not alter the half-life of FOXA1 mRNA in TE13 and KYSE450 cells, as measured by ActD-chase assays using qPCR. Data shown are mean ± s.d.; *n* = 3 independent experiments, two-tailed Student’s *t*-test. **f** and **g** SiRNA knockdown of FOXA1 (f) abolished the upregulation of PARylator by CDDP treatment (g) in TE13 and KYSE450 cells, as measured Western blotting (f) and qPCR (g), respectively, Data shown are representatives (f) or mean ± s.d. (g); *n* = 3 independent experiments, one-way ANOVA followed by Tukey’s multiple comparison test

Since PARylator promotes PARP1 activation and genome maintenance, we tested whether DNA damage regulates PARylator expression. Treatment with CDDP (0.5 µg/ml, 24 h) increased PARylator levels in TE13 and KYSE450 cells as assessed by qPCR (Fig. 6a), indicating that PARylator is upregulated in response to DNA damage. Actinomycin D (ActD, 200 ng/ml) chase experiments indicated no change in the half-life of PARylator after exposure to CDDP (Fig. 6b), suggesting that the observed upregulation is due to increased transcription rather than changes in RNA stability.

Given that FOXA1 activates PARylator transcription (Fig. 1h-n), we asked whether FOXA1 is itself induced by CDDP. Both mRNA and protein levels of FOXA1 increased in TE13 and KYSE450 cells following CDDP treatment (0.5 µg/ml, 24 h) (Fig. 6c, d). Notably, FOXA1 mRNA levels elevated without a change in its half-life, as demonstrated by qPCR through ActD (200 ng/ml) chase assays (Fig. 6e), suggesting transcriptional induction. Importantly, FOXA1 knockdown abolished the CDDP-induced increase in PARylator expression (Fig. 6f, g), supporting a model in which DNA treatment with CDDP upregulates PARylator through FOXA1‑mediated transcription.

### Inhibiting PARylator reduces the growth of ESCC and sensitizes ESCC cells to DNA-damaging therapies

**Fig. 7:**
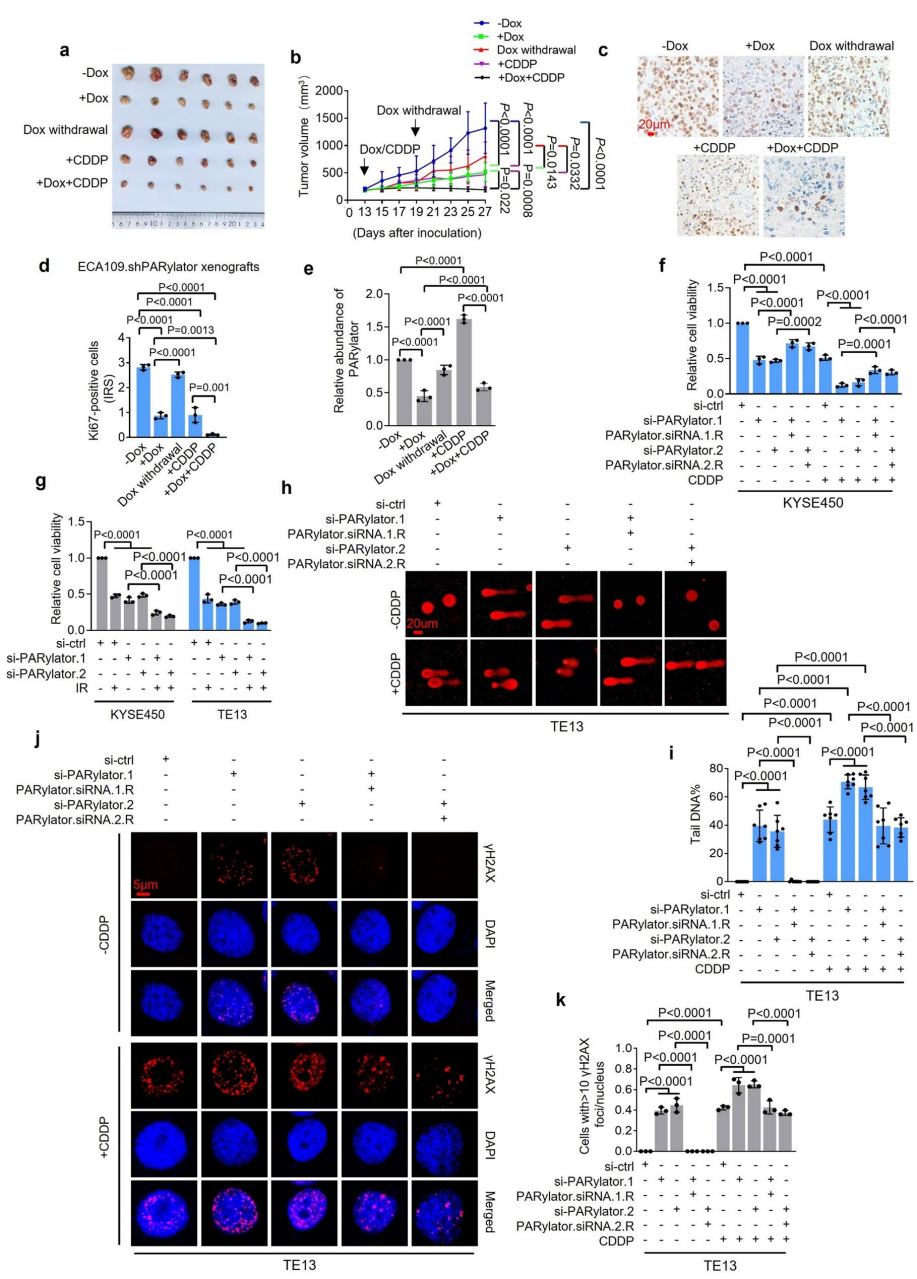
PARylator knockdown reduces ESCC growth and sensitizes ESCC cells to DNA damage. **a** and **b** Representative photographs (a) and growth curves (b) of ECA109.shPARylator xenografts in nu/nu mice (*n* = 6 tumors/group), treated as indicated. Data shown are representatives (a) or mean ± s.d. (b); one-way ANOVA followed by Tukey’s multiple comparisons test. **c** and **d** Representative microscopic photographs (c) and quantification (d) of Ki67 staining on randomly selected ECA109.shPARylator tumors (*n* = 3 tumors) from mice treated as indicated. Data shown are representatives (c) or mean ± s.d. (d); one-way ANOVA followed by Tukey’s multiple comparison test. IRS: immunoreactive score. Scale bar, 20 μm. **e** PARylator expression in representative ECA109.shPARylator xenografts (*n* = 3 tumors) from mice treated as indicated, measured by qPCR. Data shown are mean ± s.d.; one-way ANOVA followed by Tukey’s multiple comparison test. **f** Cell viability of KYSE450 cells treated with CDDP (0.25 µg/ml, 48 h), cells were subjected to: (i) Control siRNA, (ii) PARylator siRNA, or (iii) PARylator siRNA followed by rescue with an siRNA-resistant PARylator expression plasmid. Data shown are mean ± s.d.; *n* = 3 independent experiments, one-way ANOVA followed by Tukey’s multiple comparison test. **g** SiRNA knockdown of PARylator sensitized ESCC cells to IR (4 Gy, single fraction) in TE13 and KYSE450 cells, as measured by CCK-8 assays. Data shown are mean ± s.d.; one-way ANOVA followed by Tukey’s multiple comparison test. **h** and **i** Representative microphotographs (h) and quantification (i) of comet tails in TE13 cells treated with or without CDDP (0.25 µg/ml, 48 h), cells were subjected to: (i) Control siRNA, (ii) PARylator siRNA, or (iii) PARylator siRNA followed by rescue with an siRNA-resistant PARylator expression plasmid. Data shown are representatives (h) or mean ± s.d. (i); *n* = 3 independent experiments, one-way ANOVA followed by Tukey’s multiple comparison test. Scale bar, 20 μm. **j** and **k** Representative microphotographs (j) and quantification (k) of immunofluorescence staining of γH2AX in TE13 cells treated with or without CDDP (0.25 µg/ml, 48 h), cells were subjected to: (i) Control siRNA, (ii) PARylator siRNA, or (iii) PARylator siRNA followed by rescue with an siRNA-resistant PARylator expression plasmid. Data shown are representatives (j) or mean ± s.d. (k); *n* = 3 independent experiments, one-way ANOVA followed by Tukey’s multiple comparison test. Scale bar, 5 μm

We tested whether PARylator supports ESCC growth in vivo. Treatment with Dox (2 mg/kg, i.p., every other day, 7 doses) to induce PARylator knockdown significantly slowed the growth of ECA109.shPARylator xenografts in nu/nu mice (Fig. 7a, b). This slowdown was associated with reduced cell proliferation and increased apoptosis (Fig. 7c, d and Supplementary Fig. 4a, b). Upon discontinuation of Dox treatment (after 3 doses), we observed that PARylator expression recovered, which was associated with increased proliferation, decreased apoptosis, and regrowth of ECA109.shPARylator xenografts (Fig. 7a-e and Supplementary Fig. 4a, b). These results suggest that PARylator may play a role in promoting ESCC tumorigenicity in vivo.

We next asked whether PARylator modulates ESCC sensitivity to CDDP and IR. Knockdown of PARylator sensitized ESCC cells to CDDP (0.25 µg/ml, 48 h) and IR (4 Gy, single fraction), as evidenced by further reductions in viability measured using CCK8 assays (Fig. 7f, g and Supplementary Fig. 4c). This sensitization was reduced by ectopic expression of siRNA-resistant PARylator, indicating on‑target specificity (Fig. 7f, h, j, Supplementary Fig. 4c, and Supplementary Fig. 5c). Sensitization was accompanied by increased comet tail moments and augmented γH2AX and 53BP1foci, measured through IF analysis (Fig. 7h-k, Supplementary Fig. 4d-i and Supplementary Fig. 5a-d), manifesting increased DNA damage. Notably, introduction of PARylator siRNA into HET-1 A cells did not cause a significant increase in reduction in viability resulting from treatment with CDDP (Supplementary Fig. 5e), suggesting that PARylator plays a limited role in regulating DNA damage sensitivity in this esophageal epithelial cell line.

Finally, in vivo combination studies mirrored the findings observed in vitro. co-treatment with Dox (2 mg/kg, i.p., every other day, 7 doses) and CDDP (3 mg/kg, i.p., every other day, 10 doses) suppressed ECA109.shPARylator xenograft growth significantly more than treatment with either Dox or CDDP alone in nu/nu mice (Fig. 7a, b). This enhanced inhibition was associated with decreased proliferation and increased apoptosis (Fig. 7c, d and Supplementary Fig. 4a, b). Therefore, inhibiting PARylator may improve the therapeutic efficacy of DNA-damaging therapies in ESCC.

## Discussion

**Fig. 8:**
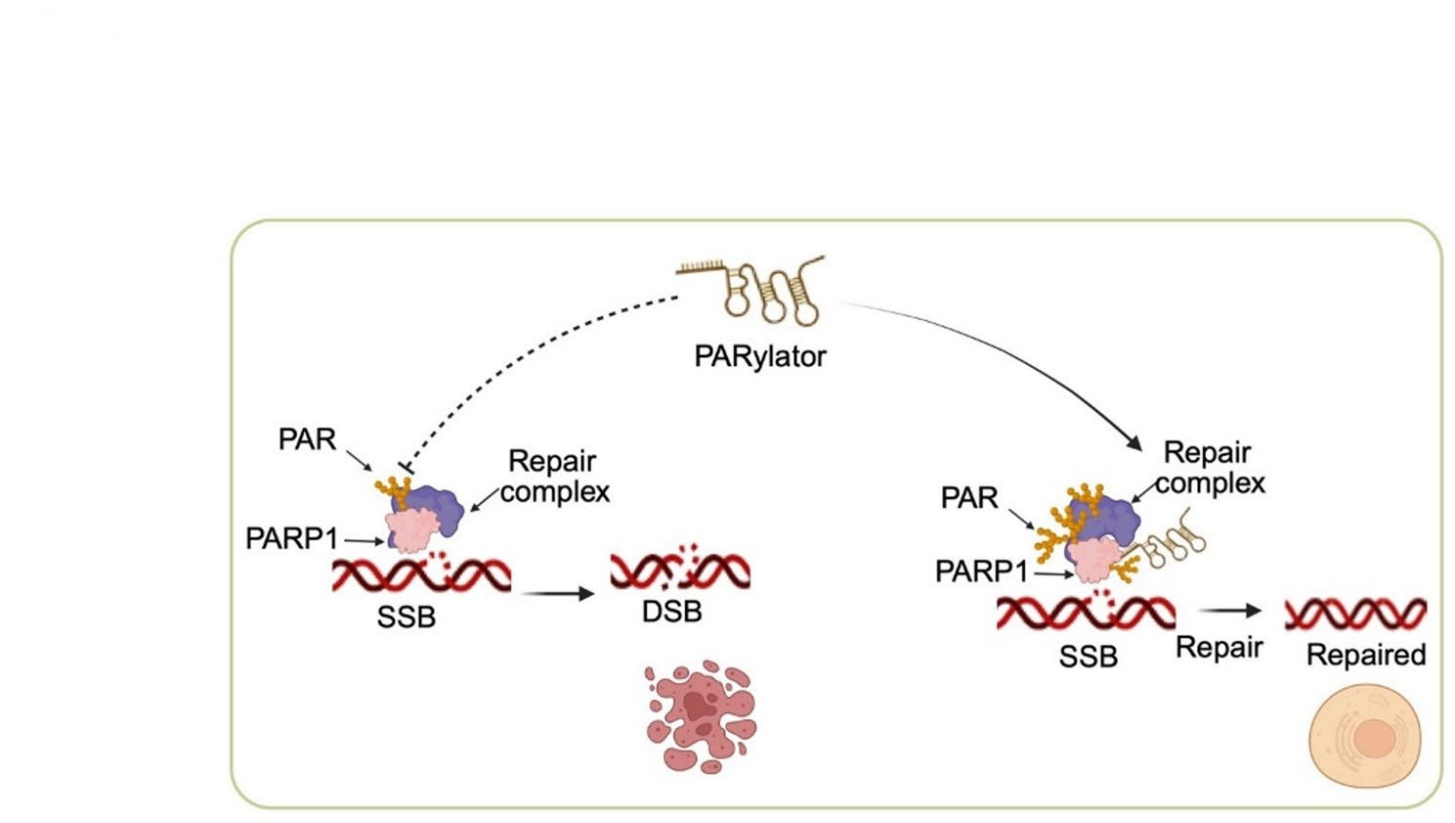
Schematic model a PARylator-mediated anti-DNA damage mechanism in ESCC. PARylator promotes PARP1 activation, facilitating SSB repair and limiting DSB accumulation, thereby protecting ESCC cells from DNA damage and supporting survival and growth

This study identifies PARylator as a DNA damage-responsive lncRNA that is frequently upregulated in ESCC through a combination of copy number gain and FOXA1-driven transcriptional activation. Mechanistically, PARylator binds PARP1 through its ZnF1 motif, facilitating PARP1 chromatin association, thus enhancing its activation and PARylation, promoting SSB repair, and reducing the accumulation of DSBs (Fig. 8). Functionally, PARylator supports ESCC cell survival and tumor growth, and its knockdown increases the sensitivity of ESCC cells to DNA-damaging therapies. These findings position PARylator as a previously unrecognized RNA cofactor of PARP1 that may protect ESCC cells against genotoxic stress through promoting SSB repair.

While previous studies have linked lncRNAs to DSB repair pathway choice or ligation efficiency [10, 31–33], PARylator operates upstream at the level of SSB sensing and early repair by potentiating PARP1. The requirement of PARP1 ZnF1 for PARylator binding, together with reduced PARP1 chromatin association and auto-PARylation upon PARylator knockdown, suggests that the RNA-protein interaction enhances the ability of PARP1 to engage DNA lesions and transition into an active state. Although PARP1 can bind structured RNAs, prior reports have variably described RNA as either a competitor for DNA binding or an allosteric modulator [34, 35]. Our data favor a cofactor model in which PARylator promotes productive PARP1 engagement with damaged DNA rather than competing for the DNA-binding interface [34, 35]. This is consistent with the observed reduction in XRCC1 recruitment and the increase in DSB surrogates when PARylator was knocked down. The rescue of function with an siRNA‑resistant wild-type PARylator but not a PARP1-binding-deficient mutant further supports the specificity of this interaction. Nevertheless, high-resolution structural studies are needed to define the precise binding interface and to test whether PARylator directly modulates PARP1 allostery or catalytic kinetics.

Importantly, we demonstrate that PARylator is itself induced by DNA damage through transcriptional upregulation mediated by FOXA1. This establishes a feedforward mechanism in which genotoxic stress increases FOXA1, which in turn elevates PARylator to promote PARP1-dependent SSB repair (Fig. 8). Given the role of FOXA1 as a pioneer factor that remodels chromatin and supports lineage transcriptional programs [36–38], its coupling to the expression of DNA repair-linked lncRNAs may exemplify a broader paradigm whereby lineage factors tune repair capacity to environmental stress [39, 40]. The predominantly nuclear localization of PARylator aligns with such a role. However, the mechanisms responsible for transcriptional upregulation of FOXA1 in ESCC cells in response to genotoxic stress require further investigation.

These mechanistic insights bear potential practical implications. By facilitating PARP1 activation in response to DNA damage, PARylator supports ESCC cell survival upon exposure to CDDP or IR. Its knockdown increased DNA damage markers, apoptosis, and treatment efficacy in vitro and in vivo, suggesting that PARylator is a tractable vulnerability. Antisense oligonucleotides or siRNA therapeutics targeting PARylator, or small molecules that disrupt the PARylator-PARP1 interaction, could be explored as potential radiosensitizers or chemo-potentiators in ESCC [41–44]. Given the clinical use of PARP inhibitors (PARPi), it will be important to determine whether PARylator modulates PARP inhibitor (PARPi) responses [10, 45]. On the one hand, PARylator inhibition may phenocopy aspects of PARP1 loss-of-function and enhance cytotoxicity to DNA-damaging agents. On the other hand, PARPi induce PARP1 trapping [14, 46], whereas PARylator facilitates the recruitment of PARP1 to chromatin. How these seemingly paradoxical processes intersect could influence trapping, repair factor assembly, and net cytotoxicity needs further investigation. PARylator abundance may serve as a biomarker to predict sensitivity to DNA-damaging and PARP1-targeted therapies.

In summary, we demonstrate that the DNA damage-responsive lncRNA PARylator is commonly upregulated by gene copy number gain and FOXA1-mediated transcription, and functions as an RNA cofactor that strengthens PARP1-dependent DNA repair and fosters ESCC cell survival under genotoxic stress (Fig. 8). Targeting PARylator may disrupt this adaptive mechanism and improve the efficacy of DNA-damaging therapies in ESCC treatment. Notably, PARylator is also upregulated in several other types of SCC, such as LUSC and CESC, but whether it plays a similar role in promoting PARP1 activation and thus protecting against DNA damage needs further study.

## Conclusions

The DNA damage-responsive lncRNA PARylator, encoded within the 3q26-q29 amplicon, plays a significant role in promoting ESCC cell survival, proliferation, and tumorigenicity by facilitating PARP1-mediated PARylation. This process enhances SSB repair, limits DSB accumulation, and confers resistance to DNA-damaging therapies. As such, targeting PARylator, either alone or in combination with DNA-damaging agents, represent a potential avenue for ESCC treatment.

However, the study faces several limitations. The use of specific cell lines and animal models may not entirely reflect the complexities of human ESCC, which might limit the general relevance of the results. Although initial evidence supports the interaction between PARylator and PARP1, detailed structural studies are needed to fully understand their binding interaction and how PARP1 activity is modulated. Furthermore, while the study suggests potential therapeutic applications, significant hurdles remain in translating these findings into clinical practice, which requires further exploration.

## Supplementary Information

Below is the link to the electronic supplementary material.

Supplementary Material 1.

Supplementary Material 2.

## Data Availability

RNA-sequencing data that support the findings of this study have been deposited in Gene Expression Omnibus (GEO) at GSE308019. The publicly available RNA sequencing data analyzed in this study were obtained from TCGA at https://xenabrowser.net/datapages/?dataset=TCGA-ESCA.star_counts.tsv&host=https%3 A%2 F%2Fgdc.xenahubs.net&removeHub=http%3 A%2 F%2F127.0.0.1%3A7222. All other underlying data for the manuscript are available upon request from the corresponding author T.L.

## Abbreviations

ESCC: Esophageal squamous cell carcinoma
lncRNAs: Long non-coding RNAs
PARP1: Poly(ADP-ribose) polymerase 1
PAR: Poly(ADP-ribose)
SSB: Single-strand DNA break
DSB: Double-strand DNA break
DDR: DNA damage response
FOXA1: Forkhead box protein A1
γH2AX: Phosphorylated histone H2AX
53BP1: P53-binding protein 1
CDDP: Cisplatin
IR: Ionizing radiation
XRCC1: X-ray repair cross-complementing 1
SOX2: SRY-Box Transcription Factor 2
eIF4G1: Eukaryotic translation initiation factor 4 gamma 1
FFPE: Formalin-fixed, paraffin-embedded
CCK8: Cell Counting Kit-8
qPCR: Quantitative PCR
RNA-seq: RNA sequencing
IHC: Immunohistochemistry
ISH: In situ hybridization
MS: Mass spectrometry
ChIP: Chromatin Immunoprecipitation
RPD: RNA pull-down
RIP: RNA immunoprecipitation
PUMA: P53 upregulated modulator of apoptosis
GSEA: Gene set enrichment analysis
DBD: DNA-binding domain
BRCT: BRCA1 C-terminus
TCGA: The Cancer Genome Atlas
LUSC: Lung squamous cell carcinoma
CESC: Cervical squamous cell carcinoma
ZnF: Zinc finger motif
ATM: Ataxia telangiectasia mutated
CHK2: Checkpoint kinase 2
H_2_O_2_: Hydrogen peroxide
