# Supplementary material for: The lncRNA PARylator promotes PARP1 activation and resistance to DNA‑damaging therapy in esophageal squamous cell carcinoma
Source: Exp Hematol Oncol. 2025 Dec 31;15:3. doi: 10.1186/s40164-025-00739-z (PMC12781723; doi:10.1186/s40164-025-00739-z)
Supplement: Supplementary file 1 — Supplementary Material 1. [file 40164_2025_739_MOESM1_ESM.pdf]

## Supplementary Figure 1

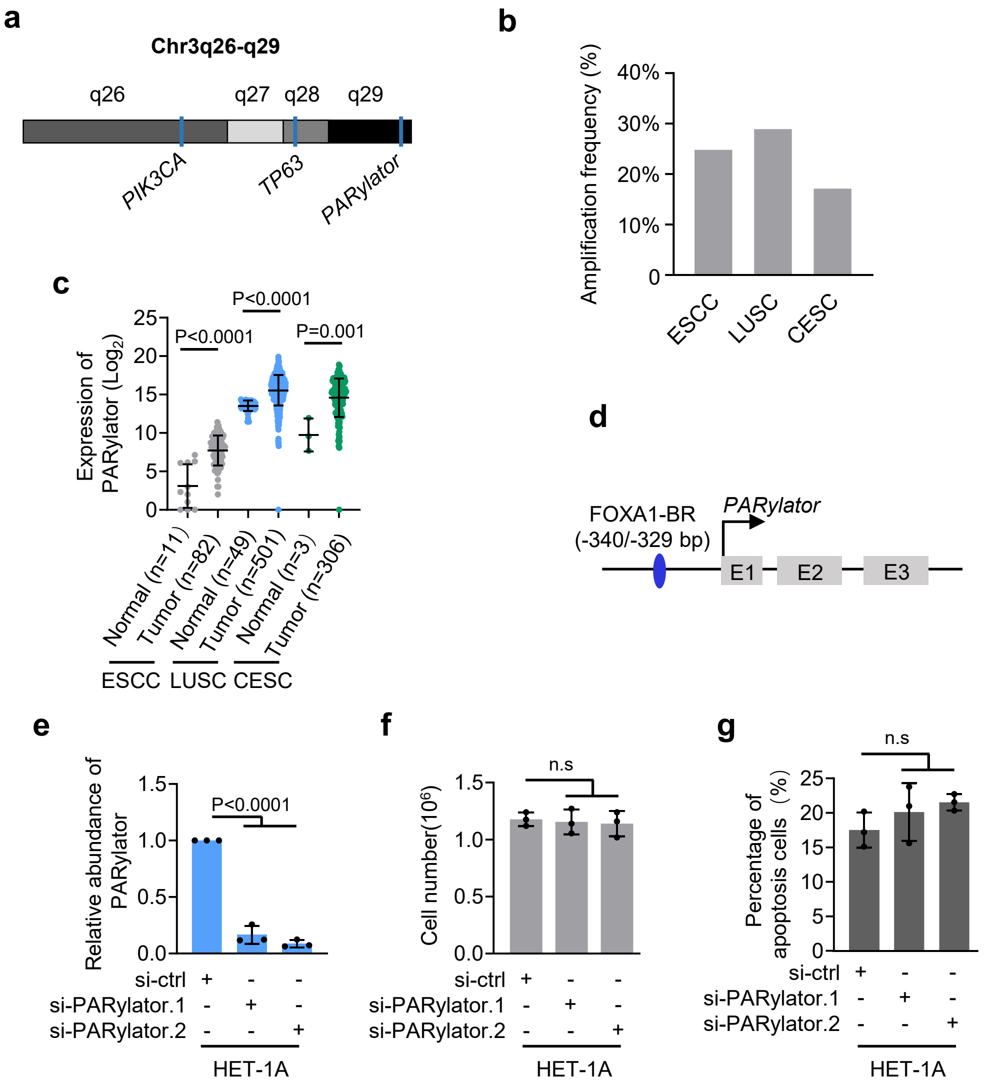


**Supplementary Figure 1. PARylator is upregulated in ESCC cells by genomic amplification and FOXA1-mediated transcription**

**(a)** Schematic illustration of the genomic localization of the *LINC00885* (*PARylator*) gene within the distal portion of chromosome 3q (3q26-q29).
**(b)** Analysis of the TCGA-ESCC, TCGA-LUSC, and TCGA-CESC datasets showing that the *LINC00885* (*PARylator*) gene is amplified in ESCC (n = 96), LUSC (n = 492), and CESC (n = 301).
**(c)** Analysis of the TCGA-ESCC, TCGA-LUSC, and TCGA-CESC datasets showing that LINC00885 (PARylator) is upregulated in ESCC (n = 82), LUSC (n = 501), and CESC (n = 306), compared with corresponding normal tissues (n = 11, 49, and 3, respectively). Data shown are mean ± s.d.; two-tailed Student's t-test.
**(d)** Schematic illustration depicting the location of the FOXA1 binding region (FOXA1-BR) at the proximal promoter of the *PARylator* gene.
**(e-g)** SiRNA knockdown of PARylator (e) did not reduce HET-1A cell viability (f) and cell apoptosis (g) as measured using qPCR (e), cell counting(f) and flowcytometry analysis(g), respectively. Data shown are mean ± s.d.; n = 3 independent experiments, one-way ANOVA followed by Tukey's multiple comparison test.

**Supplementary Figure 2**

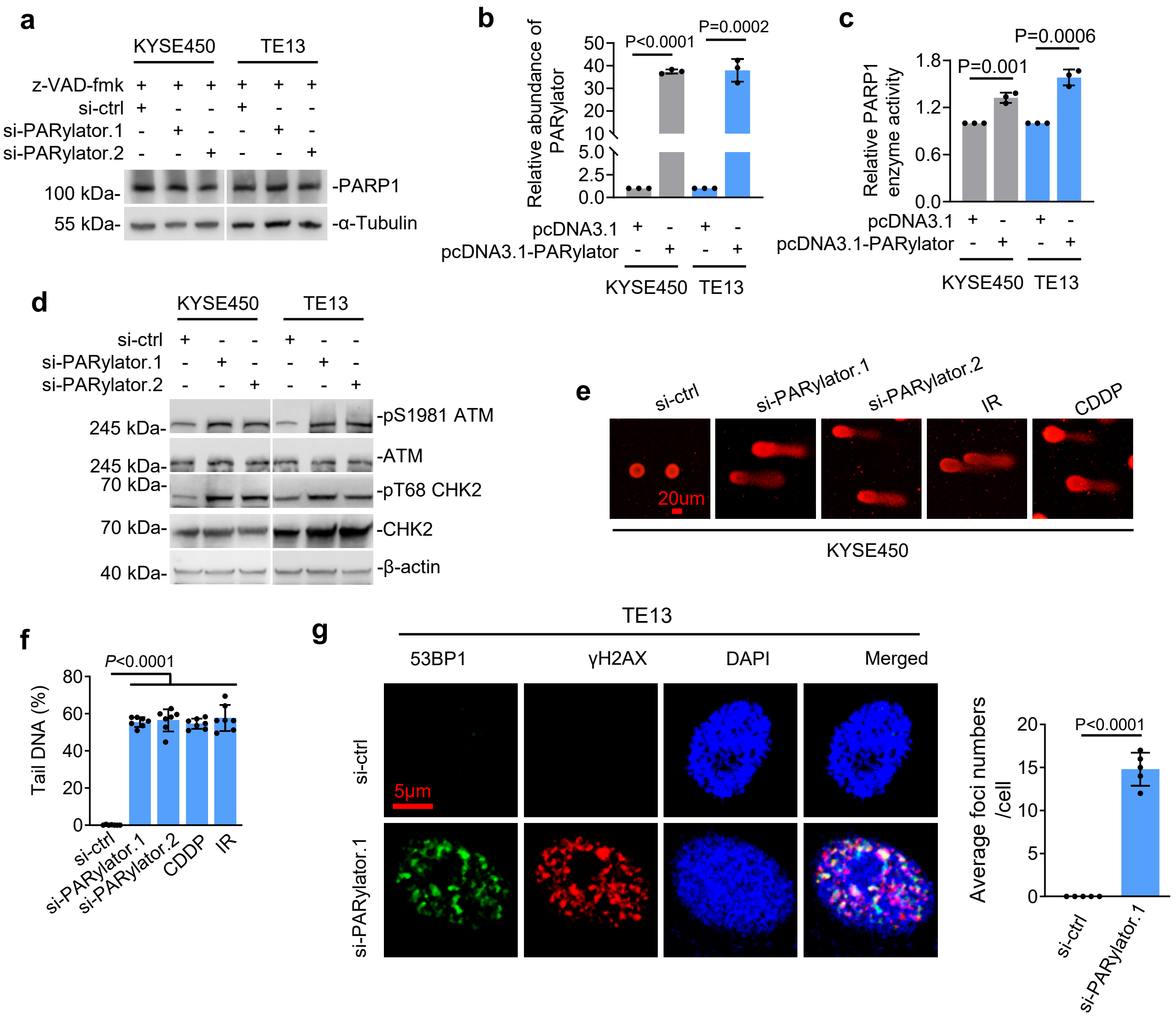


**Supplementary Figure 2. PARylator promotes PARP1 activation**

**(a)**In TE13 and KYSE450 cells pretreated with the pan-caspase inhibitor z-VAD-fmk (50 μM), siRNA knockdown of PARylator did not alter PARP1 expression levels, as determined by Western blotting. Data shown are representatives; n = 3 independent experiments.

**(b and c)** Overexpression of PARylator (b) increased PARP1 activity (c), as measured using qPCR (b) and colorimetric PARP1 activity assays (c), respectively. Data shown are mean ± s.d.; n = 3 independent experiments, two-tailed Student's t-test.

**(d)** SiRNA knockdown of PARylator increased phosphorylation of ATM and CHK2, as determined by Western blotting. Data shown are representatives; n = 3 independent experiments.

**(e and f)** Representative microphotographs (e) and quantification (f) of comet tails in KYSE450 cells with or without siRNA knockdown of PARylator. Data shown are representatives (e) or mean ± s.d. (f); n = 3 independent experiments, one-way ANOVA followed by Tukey's multiple comparison test. Scale bar, 20 μm.

**(g)** SiRNA knockdown of PARylator caused the accumulation of 53BP1 foci that colocalized with γH2AX in TE13 cells, as measured by immunofluorescence staining and quantification. Data shown are representatives; n = 3 independent experiments. Scale bar, 5 μm.

**Supplementary Figure 3**

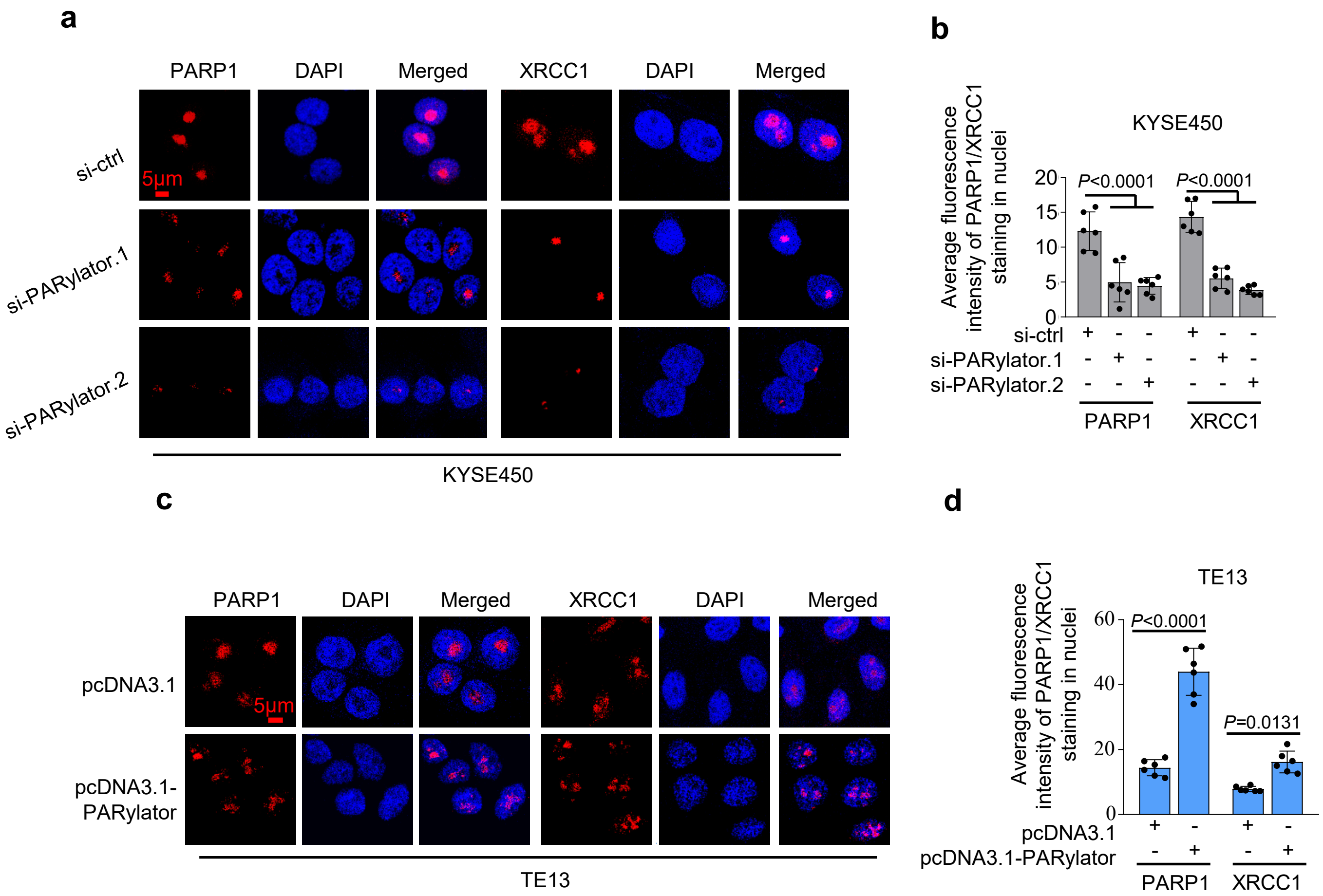


**Supplementary Figure 3. PARylator promotes PARP1 chromatin association**

**(a and b)** Representative microphotographs (a) and quantification (b) of immunofluorescence staining of PARP1 and XRCC1 under detergent pre-extraction conditions in KYSE450 cells with PARylator knockdown treated with $H_2O_2$ (200 µM, 1 h). Data shown are representatives (a) or mean ± s.d. (b); n = 3 independent experiments, one-way ANOVA followed by Tukey's multiple comparison test. Scale bar, 5 µm.

**(c and d)** Representative microphotographs (c) and quantification (d) of immunofluorescence staining of PARP1 and XRCC1 under detergent pre-extraction conditions in TE13 cells overexpressing PARylator and treated with $H_2O_2$ (200 µM, 1 h). Data shown are representatives (c) or mean ± s.d. (d); n = 3 independent experiments, one-way ANOVA followed by Tukey's multiple comparison test. Scale bar, 5 µm.

# Supplementary Figure 4

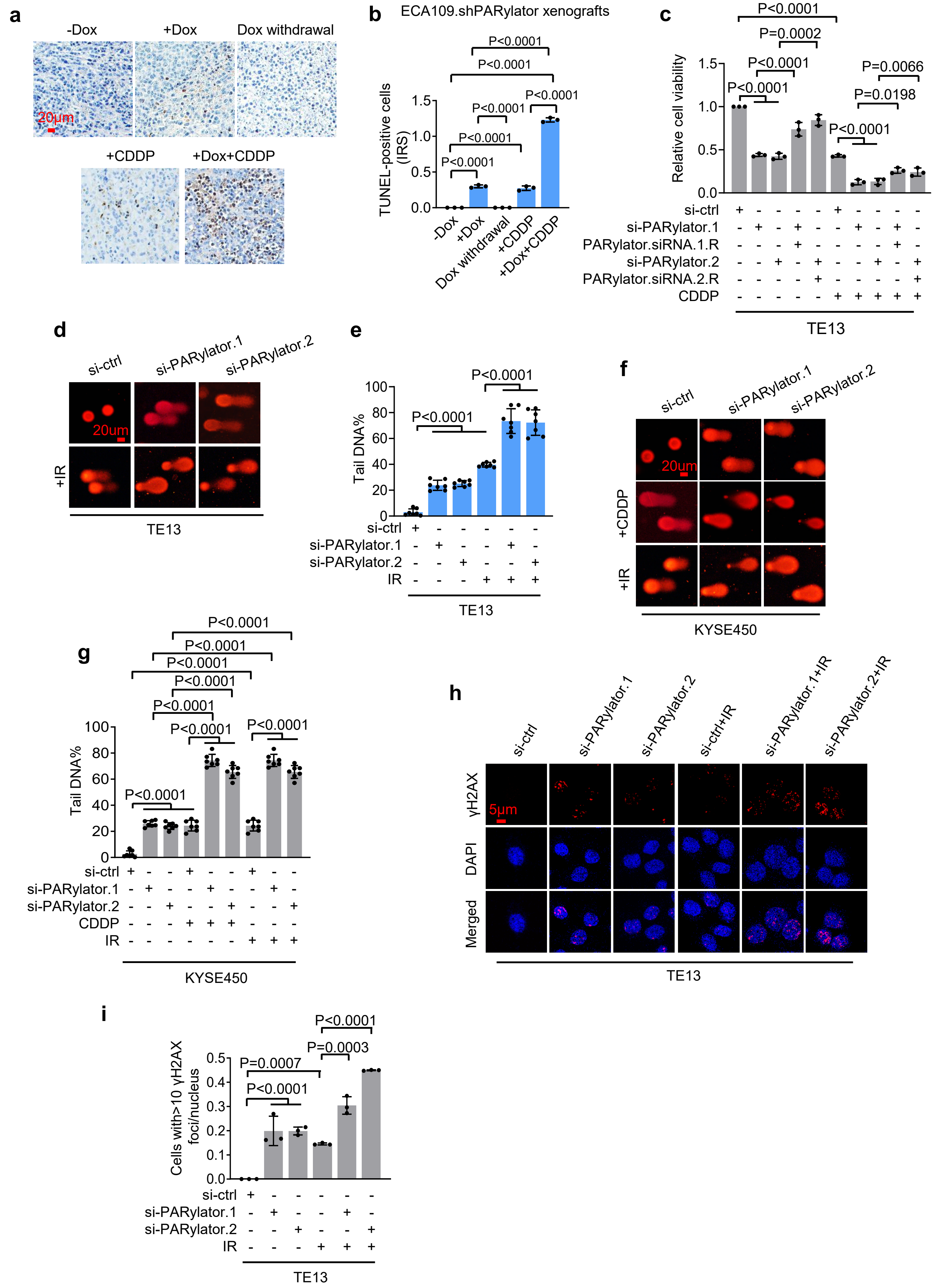

**Supplementary Figure 4. PARylator knockdown reduces ESCC growth and sensitizes ESCC cells to DNA damage**

**(a** and **b)** Representative microscopic photographs (a) and quantification (b) of TUNEL staining on randomly selected ECA109.shPARylator tumors (n = 3 tumors) from mice treated as indicated. Data shown are representatives (a) or mean ± s.d. (b); one-way ANOVA followed by Tukey's multiple comparison test. IRS: immunoreactive score. Scale bar, 20 μm.

(**c**) Cell viability of TE13 cells treated with CDDP (0.25 μg/ml, 48 h), Cells were subjected to: (i) Control siRNA, (ii) PARylator siRNA, or (iii) PARylator siRNA followed by rescue with an siRNA-resistant PARylator expression plasmid. Data shown are mean ± s.d.; n = 3 independent experiments, one-way ANOVA followed by Tukey's multiple comparison test.

**(d** and **e)** Representative microphotographs (d) and quantification (e) of comet tails in TE13 cells with or without siRNA knockdown of PARylator, followed by treated with IR (4Gy, single fraction). Data shown are representatives (d) or mean ± s.d. (e); n = 3 independent experiments, one-way ANOVA followed by Tukey's multiple comparison test. Scale bar, 20 μm.

**(f** and **g)** Representative microphotographs (f) and quantification (g) of comet tails in KYSE450 cells with or without siRNA knockdown of PARylator treated with CDDP (0.25 μg/ml, 48 h) or IR (4Gy, single fraction). Data shown are representatives (f) or mean ± s.d. (g); n = 3 independent experiments, one-way ANOVA followed by Tukey's multiple comparison test. Scale bar, 20 μm.

**(h** and **i)** Representative microphotographs (h) and quantification (i) of immunofluorescence staining of γH2AX in TE13 cells with or without siRNA knockdown of PARylator treated with IR (4Gy, single fraction). Data shown are representatives (h) or mean ± s.d. (i); n = 3 independent experiments, one-way ANOVA followed by Tukey's multiple comparison test. Scale bar, 5 μm.

# Supplementary Figure 5

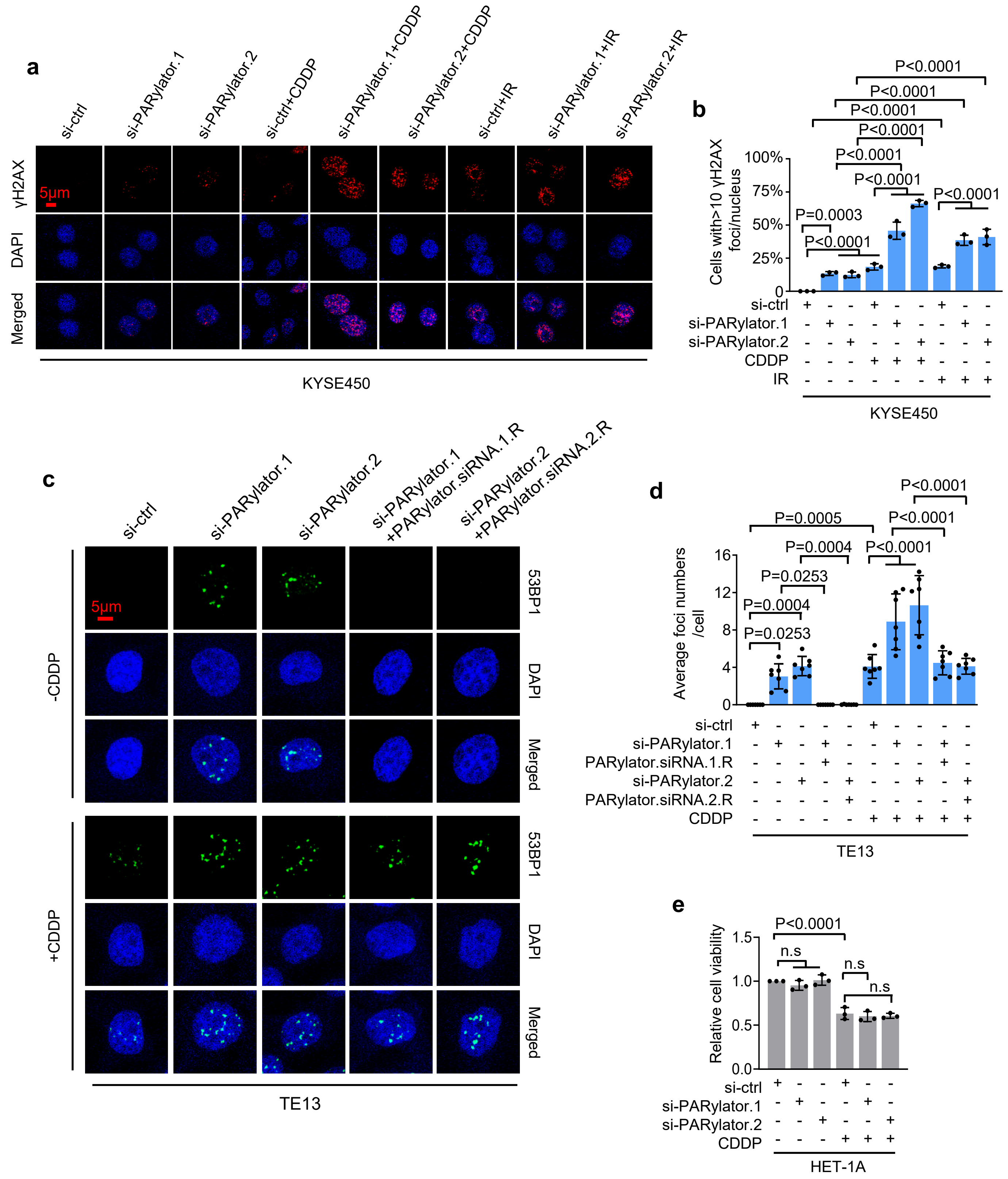

**Supplementary Figure 5. PARylator knockdown reduces ESCC growth and sensitizes ESCC cells to DNA damage**

**(a and b)** Representative microphotographs (a) and quantification (b) of immunofluorescence staining of γH2AX in KYSE450 cells with or without siRNA knockdown of PARylator treated with CDDP (0.25 μg/ml, 48 h) or IR (4Gy, single fraction). Data shown are representatives (a) or mean ± s.d. (b); n = 3 independent experiments, one-way ANOVA followed by Tukey's multiple comparison test. Scale bar, 5 μm.

**(c and d)** Representative microphotographs (c) and quantification (d) of immunofluorescence staining of 53BP1 in TE13 cells treated with CDDP (0.25 μg/ml, 48 h), cells were subjected to: (i) Control siRNA, (ii) PARylator siRNA, or (iii) PARylator siRNA followed by rescue with an siRNA-resistant PARylator expression plasmid. Data shown are representatives (c) or mean ± s.d. (d); n = 3 independent experiments, one-way ANOVA followed by Tukey's multiple comparison test. Scale bar, 5 μm.

**(e)** Cell viability of HET-1A cells with or without siRNA knockdown of PARylator treated with CDDP (0.25 μg/ml, 48 h). Data shown are mean ± s.d.; n = 3 independent experiments, one-way ANOVA followed by Tukey's multiple comparison test.
