# Supplementary material for: The lncRNA PARylator promotes PARP1 activation and resistance to DNA‑damaging therapy in esophageal squamous cell carcinoma
Source: Exp Hematol Oncol. 2025 Dec 31;15:3. doi: 10.1186/s40164-025-00739-z (PMC12781723; doi:10.1186/s40164-025-00739-z)
Supplement: Supplementary file 2 — Supplementary Material 2. [file 40164_2025_739_MOESM2_ESM.pdf]

Supplementary Tables

Supplementary Table 1. List of cell lines

Cell lines	Source	Catalogue No.
ECA109	YaJi Biological (Shanghai, China)	YS106C
KYSE30	YaJi Biological (Shanghai, China)	YS321C
HET-1A	YaJi Biological (Shanghai, China)	YS1847C
KYSE150	YaJi Biological (Shanghai, China)	YS168C
KYSE450	YaJi Biological (Shanghai, China)	YS3155C
TE13	YaJi Biological (Shanghai, China)	YS293C

Supplementary Table 2. List of antibodies

Antibody (Ab)	Catalogue No.	Company	Dilution	RRID
GAPDH Mouse pAb	60004-1-Ig	Proteintech Group (Wuhan, Hubei, China)	1:10000	AB_2107436
PARP1 Mouse pAb	66520-1-Ig	Proteintech Group (Wuhan, Hubei, China)	1:5000	AB_2881883
FOXA1 Rabbit mAb	20411-1-AP	Proteintech Group (Wuhan, Hubei, China)	1:2000	AB_10667003
Poly(ADP-ribose) mAb(10H)	ELS-ALX-804-220-R100	ENZO LIFE SCIENCES	1:400	AB_2880691
Alpha Tubulin Mouse mAb	ab7291	Abcam (Cambridge, UK)	1:5000	AB_2241126
Ki67 Rabbit pAb	27309-1-AP	Proteintech Group (Wuhan, Hubei, China)	1:10000	AB_2756525
Normal Rabbit IgG mAb	ab172730	Abcam (Cambridge, UK)	1:1000	AB_2687931
Normal Mouse IgG pAb	sc-2025	Santa Cruz Biotechnology (Dallas, TX)	1:500	AB_737182
XRCC1	21468-1-AP	Proteintech Group (Wuhan, Hubei, China)	1:5000	AB_2878865
PUMA	55120-1-AP	Proteintech Group (Wuhan, Hubei, China)	1:1000	AB_10859944
NOXA	SC-56169	Santa Cruz Biotechnology	1:500	AB_784877
Caspase3	9664	Cell Signaling Technology	1:1000	AB_3076239
FLAG	66008-4-Ig	Proteintech Group (Wuhan, Hubei, China)	1:5000	AB_2918475
Histone H3	17168-1-AP	Proteintech Group (Wuhan, Hubei, China)	1:5000	AB_10859944
ATM	ab32420	Abcam (Cambridge, UK)	1:3000	AB_725574
p-ATM (T68)	ab81292	Abcam (Cambridge, UK)	1:50000	AB_1640207

Chk2	ab109413	Abcam (Cambridge, UK)	1:1000	AB_10863751
p-Chk2 (Thr68)	2661	Cell Signaling Technology	1:1000	AB_331479
53BP1	4937	Cell Signaling Technology	1:100	AB_10694558
DyLight 488-Goat antiRabbit IgG (H+L)	BA1127	BOSTER Biological Technology (Wuhan, Hubei, China)	1:200	AB_3713475
CY3-Goat anti-Mouse IgG (H+L)	BA1031	BOSTER Biological Technology (Wuhan, Hubei, China)	1:200	AB_10890402

mAb: monoclonal antibody
pAb: polyclonal antibody
RRID: Research Resource Identifier

Supplementary Table 3. List of reagents

Reagent	Catalogue No.	Company
Doxycycline	HY-N0565B	Med Chem Express (Nanjing, Jiangsu, China)
TRIzol Reagent	15596018	Thermo Fisher Scientific (Waltham, MA)
Protease Inhibitor Cocktail	HY-K0010	Med Chem Express (Monmouth Junction, NJ)
RiboLock RNase Inhibitor	EO0384	Thermo Fisher Scientific (Waltham, MA)
4% paraformaldehyde	AR1068	BOSTER Biological Technology (Wuhan, Hubei, China)
Opti-MEM Reduced Serum Medium	31985070	Thermo Fisher Scientific (Waltham, MA)
Protease K	P1120	Solarbio Life Sciences (Beijing, China)
Cell counting kit 8	HY-K0301	Med Chem Express (Nanjing, Jiangsu, China)
DNA extraction buffer	P1012	Solarbio Life Sciences (Beijing, P.R.C)
ANTI-FLAG® M2 Affinity Gel	A2220	SigmaAldrich
PARP1 Protein, Human (sf9, His)	HY-P74652	Med Chem Express (Nanjing, Jiangsu, China)
Puromycin dihydrochloride	HY-B1743A	Med Chem Express (Nanjing, Jiangsu, China)
Prestained Protein Ladder (10 to 245 kDa)	EC0020	SparkJade (Qingdao,Shandong,China)

Supplementary Table 4. Summary of proteins that interact with PARylator detected using mass spectrometry in KYSE450 cells

No.	Entry name	Coverage	MW (kDa)	Score
1	PARP1	46	113	256.23
2	NCL	31	76.6	95.57
3	DHX36	26	114.7	67.83
4	KRT1	28	66	63.82
5	KRT10	26	58.8	47.75
6	PC	24	129.6	46.75
7	ACACA	11	265.4	45.02
8	KRT2	31	65.4	34.73
9	TOP3A	17	112.3	33.42
10	KRT14	32	51.5	26.1
11	KRT6A	20	60	22.32
12	GTF2I	13	112.3	21.42
13	KRT16	23	51.2	20.86
14	KRT9	18	62	20.64
15	HNRNPU	19	90.5	20.39
16	UPF1	9	124.3	17.96
17	KRT5	16	62.3	16.78
18	ACTB	23	41.7	13.48
19	MSH2	5	104.7	10.51
20	FBH1	6	117.6	10.44
21	MTREX	5	117.7	10.06
22	MYO1E	7	127	7.72
23	MYO1C	5	121.6	6.09
24	KRT8	6	53.7	6.04
25	KRT18	6	48	4.54
26	MYO1B	3	131.9	4.33
27	HNRNPD	7	38.4	4.25
28	HLTF	2	113.9	3.98
29	HNRNPA1	3	38.7	3.9
30	HNRNPAB	7	36.2	3.86
31	DBN1	4	71.4	3.85
32	MATR3	3	94.6	3.77
33	YBX1	10	35.9	2.53
34	XRN2	2	108.5	2.23
35	IGKV2-40	11	13.3	2.23
36	MSH3	2	127.3	2.05
37	LIMA1	3	85.2	1.99
38	PLEC	0	531.5	1.89
39	RPA1	4	68.1	1.86
40	HSPH1	1	96.8	1.81
41	ALB	2	69.3	1.79
42	HNRNPUL2	1	85.1	1.65
43	MYH16	1	128.2	0
44	OSBPL2	4	55.2	0

Supplementary Table 5. Summary of proteins that interact with PARylator detected using mass spectrometry in TE13 cells

No.	Entry name	Coverage	MW (kDa)	Score
1	PARP1	46	113	159.62
2	NCL	31	76.6	103.93
3	DHX36	26	114.7	29.37
4	HNRNPU	28	66	18.23
5	TOP3A	26	58.8	12.27
6	XRN2	24	129.6	10.07
7	KRT2	11	265.4	9.66
8	KRT6A	31	65.4	8.87
9	KRT10	17	112.3	8.07
10	KRT1	32	51.5	7.45
11	KRT14	20	60	6.5
12	KRT16	13	112.3	6.04
13	KRT9	23	51.2	4.69
14	UPF1	18	62	4.21
15	IGHMBP2	19	90.5	4.18
16	KRT5	9	124.3	4.14
17	POTEF	16	62.3	2.48
18	MSH2	23	41.7	2.36
19	HIST1H4A	5	104.7	1.82
20	TUFM	6	117.6	1.75
21	HNRNPUL2	5	117.7	1.74
22	ALB	7	127	0
23	PC	5	121.6	0
24	ZNF862	6	53.7	0
25	MVP	6	48	0
26	SUGCT	3	131.9	0
27	PRSS33	7	38.4	0

Supplementary Table 6. List of siRNAs/shRNAs

PARylator	siRNA.1/shRNA.1:<br>CCAAGATAAAGCACCAACACCTGAGGA<br>siRNA.2/shRNA.2:<br>CCCCAGTGGAATCATGGGGATTTCTTA
PARylator-siRNA.1.R	CCAATCGACCTCCAAAACACCTGAGGA
PARylator-siRNA.2.R	CCCCAGTGGAATCATGGGGATTTCTTA
FOXA1	siRNA.1: GCTCCATGAACTCCATGAA<br>siRNA.2: CCTTCAATGACTGCTTCGT

Supplementary Table 7. List of RT-PCR primers and RNA pulldown probes

qRT-PCR primers	PARylator	Forward: AGATCAAGGACTTCCCCAGG<br>Reverse: TTTCGTCTTTCAGTGTTACTAGGC
	18s rRNA	Forward: GCTTAATTTGACTCAACACGGGA<br>Reverse: AGCTATCAATCTGTCAATCCTGTC
	β-actin	Forward: GGACTTCGAGCAAGAGATGG<br>Reverse: AGCACTGTGTTGGCGTACAG
	GAPDH	Forward: GCTCTCTGCTCCTCCTGTTC<br>Reverse: ACGACCAAATCCGTTGACTC
	U6	Forward: TCGCTTCGGCAGCACATAT<br>Reverse: ATTTGCGTGTCATCCTTGC
	FOXA1	Forward: AGGCATACGAACAGGCACTGCAA<br>Reverse: GGAAGTGTTTAGGACGGGTCTGGAATA
	FOXA1-BR	Forward:TCCCTGAGTTTGATGCTTGGTTT<br>Reverse:ATTCTGGTTCCGCAGGTACAGC
in vitro transcriptio n primers	PARylator	Forward: CAGACCAGACCTCAAAACAG<br>Reverse: CTAAATGTAGTGCCATTTTCTTGAA
	PARylator-△E1	Forward:GTTCCCAAGGTTACCACCCAACC<br>Reverse: CTAAATGTAGTGCCATTTTCTTGAA
	PARylator-△E2	Forward: CAGACCAGACCTCAAAACAG<br>Reverse: CTAAATGTAGTGCCATTTTCTTGAA
	PARylator-△E3	Forward: CAGACCAGACCTCAAAACAG<br>Reverse: CTGCTGTCCTCAGGTGTTGGT
PARylator -Biotin-AS-probes		Antisense 1: TAAGAAATCCCCATGATTCCACTGGGG<br>Antisense 2: TCCTCAGGTGTTGGTGCTTTATCTTGG<br>Antisense 3: GAATCAGATTAGATCCATTCTGCTCAG
PARylator -Biotin-S-probes		Scramble 1: CCCCAGTGGAATCATGGGGATTTCTTA<br>Scramble 2: CCAAGATAAAGCACCAACACCTGAGGA<br>Scramble 3: CTGAGCAGAATGGATCTAATCTGATTC

Supplementary Table 8. Summary of clinicopathological characteristics of the cohort of 111 esophageal squamous cell carcinoma patients

Characteristics	Cases	PARylator abundance in esophageal squamous cell carcinoma (RS[1])	*P* value[2]
**Sex**	111		
Male	68	0.7551 ± 0.09032 [3]	0.0466
Female	43	0.4902 ± 0.08337	
**Age**	111		
≥65[4]	36	0.6196 ± 0.09360	0.6119
＜65	75	0.6860 ± 0.09075	
**TNM Stage**	110		
I/II	46	0.8193 ± 0.1169	0.0233
III/IV	64	0.5209 ± 0.07100	
**Histological Grade**	105		
I/II	72	0.5960 ± 0.07869	0.5318
III	33	0.6855 ± 0.1222	

[1]RS: Reactive score

[2]Two-tailed Student's *t*-test; a *P* value less than 0.05 was considered statistically significant

[3]Data shown are mean ± s.e.m.

[4]The median age of the patients in this cohort was 65
